# Assessing Molecular Mechanisms of Stress Induced Salinity Adaptation in the Juvenile Ornate Spiny Lobster, *Panulirus ornatus*

**DOI:** 10.3390/ijms262211150

**Published:** 2025-11-18

**Authors:** Eleanor L. Spencer, Quinn P. Fitzgibbon, Susan Glendinning, Courtney L. Lewis, Thomas M. Banks, Andrew J. Trotter, Tomer Ventura, Gregory G. Smith

**Affiliations:** 1 Institute for Marine and Antarctic Studies (IMAS), University of Tasmania, Private Bag 49, Hobart, TAS 7001, Australia; 2 Centre for Bioinnovation, University of the Sunshine Coast, Maroochydore, QLD 4556, Australia; sglendinning@usc.edu.au (S.G.);; 3 School of Science, Technology and Engineering, University of the Sunshine Coast, Maroochydore, QLD 4556, Australia

**Keywords:** *Panulirus ornatus*, transcriptomics, salinity, osmoregulation, qPCR, transporters, channels

## Abstract

*Panulirus ornatus*, the ornate spiny lobster, is a stenohaline weak hyper-osmoregulator, yet its osmoregulatory response to salinity stress remains poorly understood. This study investigated six osmoregulatory genes—Na^+^/K^+^-ATPase (*nka*), V-type H^+^-ATPase (*vhe*), Na^+^/HCO_3_^−^ exchanger (*nbc*), Na^+^/K^+^/2Cl^−^ co-transporter (*nkcc*), Na^+^/H^+^ exchanger (*nhe*), and carbonic anhydrase (*ca*)—in juvenile gills exposed to 25 ppt, 34 ppt (control), and 40 ppt salinities during acute (48 h) and chronic (>38 d) phases. Transcriptome analysis revealed that all genes were unresponsive following either 25 ppt or 40 ppt salinity acute exposure. However, *nkcc* showed a tendency toward for upregulation under 25 ppt salinity during acute exposure. Additionally, glutathione S-transferase and putative ferrous reductase 1 were upregulated under 25 ppt salinity, suggesting increased metabolic demand. In contrast, glutathione peroxidase and an ammonia transporter were upregulated in 40 ppt salinity, indicating protein catabolism. Quantitative PCR confirmed *nkcc*- and *nka* upregulation under chronic 25 ppt salinity. *Vhe*, *nbc*, *nhe* and *ca* showed no response, and 40 ppt salinity did not affect the six target genes. These findings suggest *P. ornatus* relies on *nkcc*- and *nka*-mediated ion transport and lacks mechanisms to tolerate high salinity, resulting in reduced growth and survival. These findings define optimal salinity range for aquaculture (25–34 ppt), highlighting the need to avoid high-salinity stress in lobster water quality management

## 1. Introduction

Salinity stress poses a significant challenge for the development of near-shore and inland aquaculture [1]. Euryhaline species that are strong osmoregulators—such as Pacific white shrimp (*Litopenaeus vannamei*)—can tolerate a wide range of salinities, making them relatively easy to culture in inland waters with low salinity [2]. In contrast, stenohaline species, like the European lobster (*Homarus gammarus*), have a narrow salinity tolerance range, which limits their potential for culture outside of controlled environments such as recirculating aquaculture systems [3]. Therefore, clearly defining and maintaining optimal salinity conditions is critical when developing aquaculture practices, especially for stenohaline species. This is vital to reduce mortality, promote sustainability, and support the successful cultivation of sensitive crustaceans like the ornate spiny rock lobster (*Panulirus ornatus*) [4].

The aquaculture of *P. ornatus* is a growing industry, traditionally carried out by wild capture of early life stages, followed by grow out in sea-rafts and in nursery ponds in Southeast Asia, particularly in Vietnam (1500t in 2020) [5,6,7]. However, innovative land-based hatcheries and grow-out facilities are now being developed in countries such as Indonesia, Vietnam, and Australia [8,9]. In these tropical regions, lobsters reared in pond-based, or near-shore culture are exposed to extreme climates, such as prolonged heat waves or monsoon-like rains, which affect the salinity levels in their culture environments [1]. For instance, salinity levels in lobster culture ponds in Australia have been observed to range between 24.9 and 38.1 ppt [10]. These changes can persist for hours to days in acute cases, or for several weeks to months in chronic conditions. Adult *P. ornatus* are more commonly found in fully marine environments such as coral reefs; it has also been observed near shore [11,12]. However, following their open-ocean *phyllosoma* stage, wild juveniles often settle in shallow coastal areas and estuaries after metamorphosing into benthic juveniles, where salinity can drop below 35 ppt due to freshwater runoff [13]. In a previous study, reduced growth in *P. ornatus* was linked to culture in salinities below ambient levels, although survival remained unaffected [4]. It is likely that osmoregulation diverted metabolic energy from growth to maintaining homeostasis, contributing to these declines in this species, as was previously observed in the black tiger prawn, *Penaeus monodon* [4,14]. While the field of ionic regulation in crustaceans is well-studied for species such as brachyurans, current knowledge is limited regarding the salinity tolerances and associated molecular factors that govern adaptation and survival for spiny lobsters, including *P. ornatus*. To date, only three studies have investigated the physiological responses and effects of salinity stress on juvenile *P. ornatus*, and none have explored the molecular mechanisms underlying salinity acclimation [4,13,15].

The gills of crustaceans are crucial for respiration, excretion and osmotic control, relying on isosmotic intracellular volume regulation and anisosmotic extracellular osmoregulation [16,17]. Below 26 ppt, osmoregulators activate mechanisms of anisosmotic extracellular regulation to stabilise haemolymph osmotic and ionic concentrations, involving several ion-transporting proteins and enzymes [17]. Crustacean gills use various ion pumps and channels to manage ionic imbalances during salinity changes. Ion pumps, which are metabolically demanding, consume energy via processes like ATP hydrolysis, leading to an energy deficit that reduces somatic growth [18,19,20]. Importantly, these ion pumps can produce the electrochemical gradients required for ion transport via channels during osmoregulation. *P. ornatus* functions as a weak hyper-osmoregulator, maintaining haemolymph that is isosmotic with the external medium, at salinities between 25 and 34 ppt [4,15]. However, when exposed to salinities below 25 ppt, they demonstrate some weak osmoregulatory capacity [15]. However, moult stage significantly affects the ability of lobsters to survive under low-salinity conditions, with pre-moulting individuals exhibiting a 50% mortality rate at 20 ppt following 48 h of exposure [15]. These findings, alongside previous literature, suggest that *P. ornatus* can tolerate chronic exposure a salinity range between 25 and 40 ppt [15]. The mechanisms of osmoregulation in *P. ornatus* are not well-understood; however, in other crustaceans, ion transport proteins and enzymes in gill epithelium, such as Na^+^/K^+^-ATPase (NKA), V-type H^+^-ATPase (VHE), Na^+^/HCO_3_^−^ exchanger (NBC), Na^+^/K^+^/2Cl^−^ co-transporter (NKCC), Na^+^/H^+^ exchange protein (NHE), and carbonic anhydrase (CA), play significant roles in ion transport [21,22,23]. During salinity stress, osmotic pressure in the haemolymph is balanced by the uptake or secretion of Na^+^ and Cl^−^ in the gill epithelia [24]. Consequently, changes in external osmolarity are expected to alter the expression levels of six key genes which manage cellular osmotic and ionic stress [25]. To support salt removal during changing ambient conditions, transmembrane protein expression adjusts to meet physiological demands [26]. Together, these ion transporters and channels facilitate salt uptake in hypo-osmotic media and likely contribute to salt secretion in hyper-osmotic media, thereby compensating for passive diffusive ion fluxes [23,27,28,29].

In crustacean gills, NKA is a transmembrane protein localised to the basolateral membrane of the gill epithelia [30,31], that pumps Na^+^ out of the cytoplasm of the epithelial cell and into the haemolymph in exchange for K^+^ [32,33,34]. Na^+^/K^+^-ATPase (NKA), the most extensively studied ion transporter in osmoregulatory stress of aquatic crustaceans [21], functioning as the main driver for transepithelial movement of monovalent ions across the branchial epithelium [19,23]. The exchange of K^+^ ions for Na^+^ ions is facilitated by the energy produced during the hydrolysis of ATP to ADP, a metabolically demanding process [19]. As a key component of osmoregulation, NKA actively transports ions during low-salinity exposure to counteract their passive loss, thereby helping to maintain osmotic balance [25].

In contrast, the Na^+^/K^+^/2Cl^−^ cotransporter (NKCC) is predominantly a passive ion transporter that has been studied in far fewer crustaceans to date. The NKCC relies upon the sodium and potassium gradients established by the NKA for ion transport to occur, and is a class of membrane proteins that transport Na, K and Cl ions into and out of cells including gill epithelial cells [35]. NKCC may play different roles in ion absorption or secretion depending on its function and cell membrane localisation and its activity can be modulated by environmental salinity [36]. The NKCC mediates the co-transport of the ions at a binding stoichiometry of 1Na^+^:1K^+^:2Cl^−^ per transport [37] and is characterised as being electroneutral [35]. The Na^+^/K^+^/2Cl^−^ cotransporter occurs in two major isoforms in vertebrates: NKCC1 and NKCC2; however, there appears to be only a single copy in crustaceans NKCC [38], which is found in nonpolarized cells, where it regulates cell volume [39], as well as in certain secretory epithelia, where it is thought to drive net transport of fluid and electrolyte transport [38]. The ion-transporting NKCC gene of the Japanese gazami crab, *Portunus trituberculatus* was found to encode 1055 amino acids containing conserved AA-permease and SLC12 motifs [37]. SLC12 motif refers to the electroneutral cation-chloride cotransporter gene family.

It is well-established that external salinity can influence extracellular acid–base status in brachyuran decapod crustaceans [40,41,42]. Moreover, mechanisms which are associated with acid-based balance have been known to contribute to salinity adaptation in crustaceans [17,21,42]. Proteins involved with both acid–base balance and osmoregulation in branchial cells include the V-type ATPase (VHE) and carbonic anhydrase (CA).

VHE actively participates in ion uptake across the gill epithelium of freshwater-tolerant crustaceans and plays a critical role in acid–base balance and nitrogen excretion [23,43,44], whilst assisting with maintaining the electrochemical gradient across the cell membrane to facilitate the passive movement of Na^+^ or Cl^−^ in or out of the cell [21]. V-type H^+^-ATPase (VHE) controls Na^+^ movement by increasing the intracellular concentration of protons in the gill cells, increasing the electrochemical gradient which facilitates the movement of Na^+^ out of the branchial cells [45]. This mechanism is particularly important during osmoregulation under low-salinity or freshwater conditions, where passive ionic loss poses a significant challenge. Similarly to NKA, VHE comprises two subunits, alpha and beta. The beta subunit of VHE is predicted to play a role in osmoregulation [44], whilst in NKA, it is the alpha subunit which controls this process [46].

The CA is not involved in the direct movement of ions; however, it provides support for the ion-transporting mechanisms within the gill. Carbonic anhydrase (CA) catalyses the hydration of CO_2_, producing H^+^ and HCO_3_^−^ ions needed to support Na^+^/H^+^ and HCO_3_^−^/Cl^−^ exchange, respectively [23,47,48]. There are several isoforms of CA, which can be found either in the cytoplasm of crustacean gills or bound to the membrane. The expression of the two most prominent isoforms, both the cytoplasmic and the membrane-bound glycolytic CA are considered to be influenced by osmotic salinity change. The cytoplasmic isoform is directly involved in osmotic and ionic regulation [21,47].

Two other proteins involved with ion regulation in crustacean gills include the NHE and NBC [49,50]. In euryhaline marine species (strong osmoregulators), the initial steps in the ion uptake process involve electroneutral apical Na^+^ uptake via NHE and apical Cl^−^ uptake via Cl^−^/HCO_3_^−^ exchanger [51]. The Na^+^/H^+^ exchanger (NHE) belongs to a superfamily of cation/proton antiporters and is a member of the solute carrier (SLC) 9A family [52]. Through the transport of Na^+^ in exchange for H^+^ (1:1 stoichiometry), NHE has many roles in the cells, including regulation of ionic and pH balance, cell volume regulation and other osmoregulatory processes [53].Currently, nine isoforms of NHE have been identified. While, there is little information on the specificity of the isoform roles within crustaceans, NHE3 has been found to mediate Na^+^ absorption and H^+^ secretion in humans, where it is present in the Na^+^ absorptive cells of the mammals [52]. NHE—like Na^+^/K^+^-ATPase (NKA)—likely contributes to maintaining haemolymph osmotic pressure during periods of low-salinity stress, when passive ion loss is particularly pronounced.

The sodium bicarbonate cotransporter (NBC) is an integral membrane ion transporter that can transport HCO_3_^−^ (or a related species, such as CO_3_^2−^) across the plasma membrane in the same instance as Na^+^. This cotransport mechanism contributes to the maintenance of the electrochemical gradient, thereby supporting Na^+^ movement across the cell membrane—an essential process for osmoregulation under both high- and low- salinity stress. The SLC4 transport-related family, also known as the bicarbonate transport-related family [54], can be divided into three groups: (1) Cl^−^/HCO_3_^−^ (anion’) exchangers (AEs); (2) Na^+^/HCO_3_^−^ cotransporters (NBCs); and (3) Na^+^-driven Cl^−^/HCO_3_^−^ exchangers (NDCBEs) [55]. Knowledge of the NBC transporter in crustaceans is still lacking, with most understanding derived from vertebrates. Much of the research focuses on just the anion Cl^−^/HCO_3_^−^ exchangers in relation to pH and salinity, but not the NBC. Cai, Chen, Ren, Huang, Jiang, Gao, Huo and Hu [55] found in the Pacific white shrimp, *L vannamei*, that the NBC protein contains two functional domains (Band_3_cyto and HCO3_cotransp). High expression of the NBC in the gills in other crustacean species, including *L.vannamei*, shows that it is likely involved in osmoregulation and/or pH regulation [55].

The effects of both acute- (48 h) and chronic-term (75 d) salinity change on *P. ornatus* survival, growth and physiological response, has been well-studied in the literature [4,15]. However, no studies have focused on the function of ion-transporting-related genes in response to salinity challenges in *P. ornatus*, nor how these mechanisms may change during a different duration of exposure. Furthermore, all previous research on salinity tolerance on *P. ornatus* has focused on juvenile lobsters and not adults [4,13,15]. The present study aims to elucidate the potential roles of six ion transport-related genes through transcriptomic analysis of juvenile lobsters exposed acutely to different salinities to simulate a short-term exposure scenario. In addition, to examine the situation that may occur following a more prolonged rainfall event, or alternatively, an extended exposure to high salinity, analysis of gene expression following chronic salinity exposure was carried out. The overall aim was to understand the ion-regulatory mechanisms that would enable *P. ornatus* juveniles to maintain homeostasis in different salinities, similar to those which may occur incidentally during culture or under natural circumstances.

## 2. Results

### 2.1. Chronic Salinity Parameters

**Figure 1:**
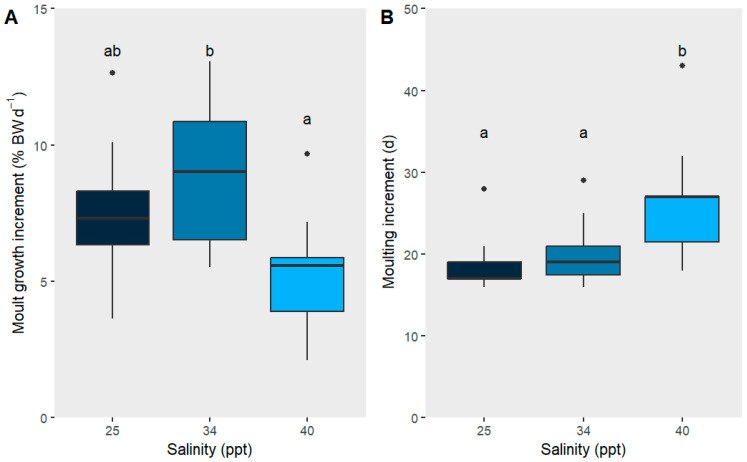
(**A**) Moult growth increment (J8–J9, %BWd^−1^); (**B**) moulting increment (J8–J9, days) of juvenile *P. ornatus* after exposure to salinities: 25, 34, and 40 ppt between instar stages 7–9 (approx. 38 days, chronic exposure; *n* = 10, 12, 11). Boxplots show the median (horizontal line), interquartile range (box), and data spread (whiskers). Dots outside the whiskers represent outliers. Significant differences between treatments are indicated by different letters (*p* < 0.05).

Salinity had a significant effect on J8–J9 growth (moult growth increment) (F_2,33_ = 7.267, *p* < 0.01; Figure 1). At 40 ppt, the juvenile lobsters exhibited reduced growth and took longer to moult compared with those in the control salinity of 34 ppt (F_2,33_ = 6.823, *p* < 0.01; Figure 1), with those at 40 ppt taking an additional 6 days to moult compared with those at 34 ppt (Table 1). Survival was similar between all three treatments (83–100%; Table 1). During the experiment, three mortalities occurred: two in the 25 ppt treatment, both related to moulting, and one in the 40 ppt treatment, unrelated to moulting. Final wet weight (WW), carapace length (CL), WW or CL gain were not significantly influenced by salinity treatments (Table 1).

**Figure 2:**
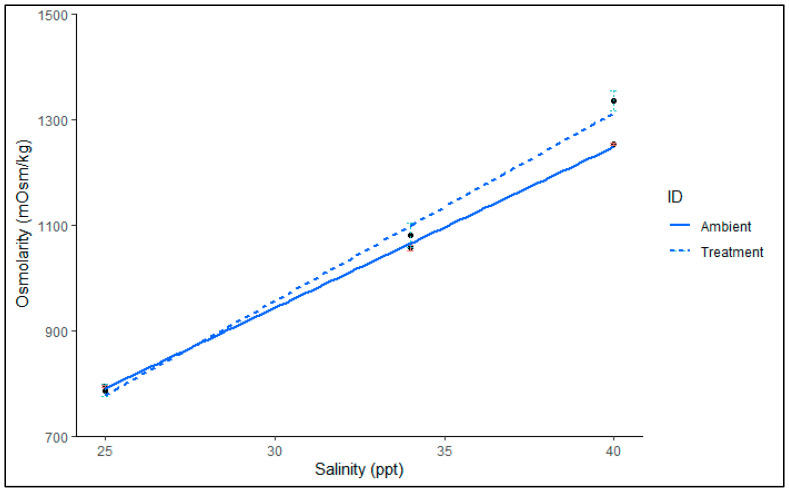
Haemolymph osmolarity (mOsm kg^−1^) of *P. ornatus* juveniles after chronic (2-moult, approx. 38 days) cycles exposure to different salinities 25, 34 and 40 ppt (dotted line) against ambient water osmolarity (solid line). Mean (±SE), with linear regression: y = poly(x, 3). Polynomic model was fitted to observed data, R^2^ > 0.97. Treatment groups, *n* = 11, 12, 10. Ambient (number of tanks sampled, *n* = 3, 3, 3). Error bars represent ±SE: green for Treatment, red for Ambient.

There was no significant association between salinity and ID (F_2,49_ = 1.01, *p* = 0.37). Salinity significantly impacted haemolymph osmolarity (*F*_3,49_ = 139.89, *p* < 0.001). There was no significant difference between ID in relation to osmolarity (*F*_3,49_ = −2.216, *p* = 0.47). However, visual analysis indicated that the haemolymph osmolarity of juvenile *P. ornatus* exposed to 40 ppt deviates from ambient salinity (Figure 2), suggesting that osmolytes were regulated to concentrations above those in the external medium. In contrast, the haemolymph of *P. ornatus* treated with 25 ppt and 34 ppt appeared to be isosmotic with the ambient salinity.

### 2.2. Transcriptome Sequencing of Panulirus ornatus Acutely Exposed Gills

**Figure 3:**
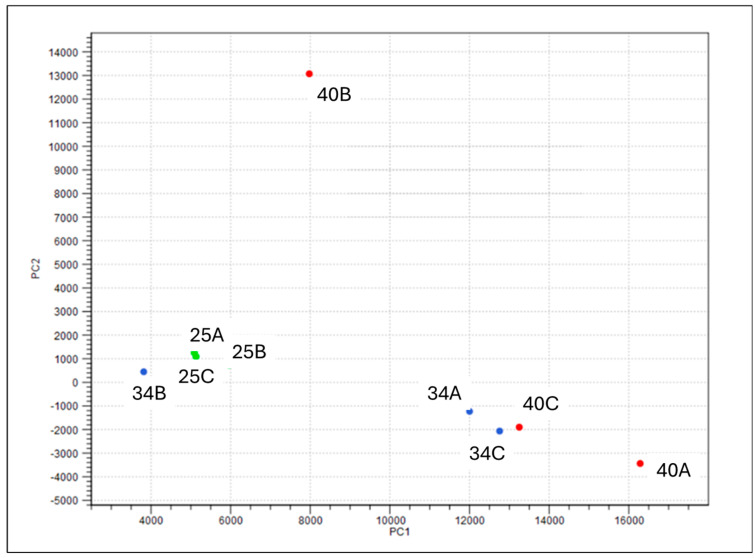
Principle component analysis (PCA) of nine *P. ornatus* gill transcriptomes after acute exposure to one of three salinity treatments for 48 h. Three 40 ppt (40A, 40B, and 40C; red), three 34 ppt (34A, 34B and 34C; blue) and three 25 ppt (25A, 25B, and 25C; green). 40B was excluded from further statistical analysis due to high variability in both PC1 and PC2 axes compared with the other high salinity animals.

A de novo transcriptome assembly of sequencing reads from three gills (one from each acute exposure salinity treatment) generated a total of 45,818 transcripts. When considering the six genes of interest, transcripts in the 40B gill sample showed markedly higher expression compared to other samples in the high-salinity treatment, with expression levels routinely exceeding 1000-fold, as highlighted in the initial DEG analysis (Figure S1, Supplementary Materials). A principal component analysis (PCA) plot of the entire transcriptome indicated that the 40B gill sample indeed varied considerably compared to the other high-salinity samples in both the PC1 and PC2 axes (Figure 3; PCA). Given that *nka* expression is typically highest in crustaceans during the pre-moult stage [56], it is suspected that the 40B individual was in early pre-moult, and thus, would have a markedly different gene expression profile compared to the intermoult samples in the high-salinity group. Therefore, to prevent overestimation of expression sample, 40B was not included in subsequent differential expression analysis and statistical analyses. Due to the limited number of replicates under 40 ppt conditions, the transcriptome analysis presented here should be considered a preliminary investigation into the expression patterns of the six genes of interest following acute salinity exposure. This analysis also includes an initial examination of differentially expressed genes (DEGs) under 40 ppt salinity. Additionally, transcriptomic data were used to identify and confirm the sequences of the six target genes investigated following chronic salinity exposure.

#### 2.2.1. Differential Expression of Genes That Occurred Following Acute Salinity Acclimation: Preliminary Analysis

**Figure 4:**
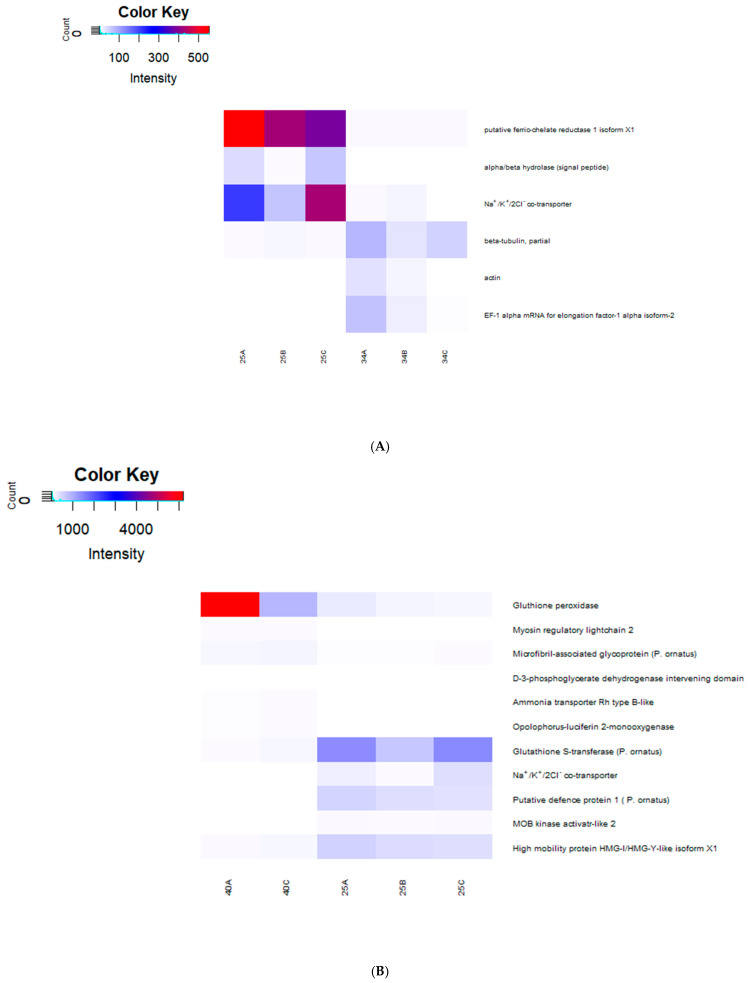
Exploratory transcriptome analysis heatmap of gills exposed to three salinity treatments (25, 34 and 40 ppt) in juvenile (instar 7–9) *P. ornatus* for 48 h (acute exposure). Differentially expressed genes only are shown (FDR < 0.05, fold-change > 2). (**A**): 25 ppt vs. 34 ppt salinity. (**B**): 25 ppt vs. 40 ppt)salinity. 25 ppt: *n* = 3, 34 ppt: *n* = 3 and 40 ppt: *n* = 2, H). The intensity of the transcript expression, represented as RPKM, are displayed as colours ranging from white (lowest expression) to dark red (highest expression).

The salinity treatments were compared to one another by determining differentially expressed genes (DEGs) following acute exposure to 25 ppt, 34 ppt or 40 ppt salinity. The analysis indicated six genes were downregulated and five were upregulated in low salinity compared with the control salinity, and one gene was upregulated in high salinity compared with the control. Comparisons between the low- and high-salinity groups were included for completeness: 13 genes were upregulated, and 13 genes downregulated in low salinity compared with high salinity. The 38 DEG transcripts were annotated, and their expression was mapped across the salinity treatment in the form of a heat map (Figure 4).

Of the 38 DEGs, 17 could be annotated, while the remaining 21 were not found to have any significant match against the NCBI database. Two transcripts upregulated in 25 ppt condition were annotated as glutathione S-transferase (GST) and putative ferrous reductase 1 isoform X1 (Figure 4A). Following 40 ppt exposure, a gene putatively coding for glutathione peroxidase (GPx) and an ammonia transporter were upregulated (Figure 4B). Other transcripts were annotated to the closest protein match in other species (Figure 4). One of the six genes of interest, NKCC, showed higher expression in gills exposed to 25 ppt salinity compared to those exposed to 40 ppt or 34 ppt (annotation and results from DEG analysis can be seen in Table S2), although due to a sample size of two for the 40 ppt group, the results should be considered tentative.

#### 2.2.2. Transcriptomic Expression of Genes of Interest

For each gene of interest, putative transcripts were identified in the *P. ornatus* assembly, and maximum likelihood trees were used to classify the six sequences alongside those from other decapods species to provide more confidence in their identification. These transcripts resolved into a single copy of *nka*, *nhe*, *ca*, *vhe*, *nkcc*, and *nbc*, with trees detailed in the Supplementary File, Figures S2–S7.

**Figure 5:**
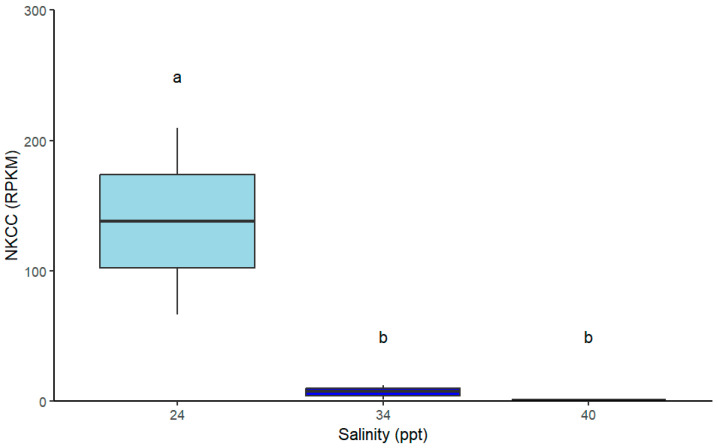
Preliminary analysis of total expression (reads per kilobase per million reads; RPKM) of Na^+^/K^+^/2Cl^−^ co-transporter transcript from *P. ornatus* juvenile gills following a 48 h salinity exposure (25, 34 and 40 ppt). 25 ppt: *n* = 3, 34 ppt: *n* = 3, and 40 ppt: *n* = 2. Boxplots show the median (horizontal line), interquartile range (box), and data spread (whiskers). Significant differences between treatments are indicated by different letters (*p* < 0.05). Expression level of genes of interest in the gills following chronic salinity acclimation.

Total expression of all genes of interest (*nbc*, *ca*, *nka*, *nhe*, *vhe* and *nkcc*) were unaffected by salinity level (Table 2. For NKCC, however, there is a clear trend which shows upregulation of this gene in the 25 ppt salinity condition, though this was not statistically significant (*p* > 0.05; Figure 5). This observed trend is indicative of some osmoregulatory mechanism in acute salinity stress but requires further validation with balanced replicate numbers. Indeed, the DEG analysis between the 34 ppt and 25 ppt salinity conditions shows that *nkcc* was significantly differentially expressed (Figure 4).

After identifying that only one of the six genes of interest was upregulated following the exploratory transcriptomic analysis of animals kept at 25 ppt for a short period of time, the same six genes were examined for any changes in expression following chronic exposure.

**Figure 6:**
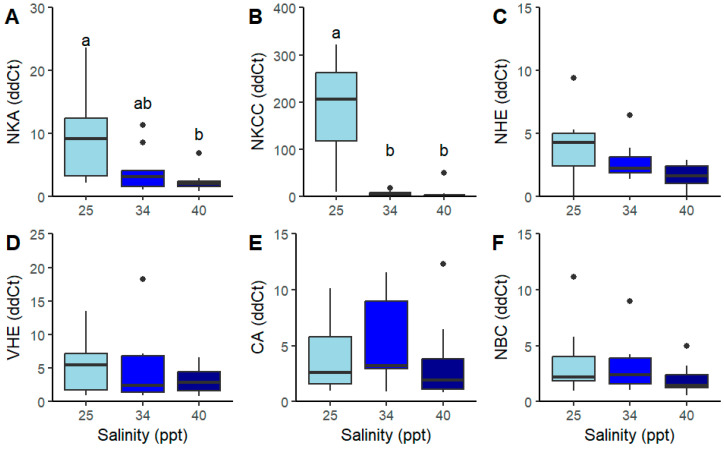
Relative expression using qPCR analysis (delta–delta cycle threshold; ddCt) of (**A**) Na^+^/K^+^-ATPase (*nka*), (**B**) Na^+^/K^+^/2Cl^−^ co-transporter (*nkcc*), (**C**) Na^+^/H^+^ exchange protein (*nhe*), (**D**) V-type H^+^-ATPase (*vhe*), (**E**) carbonic anhydrase (*ca*) and (**F**) Na^+^/HCO_3_^−^ exchanger (*nbc*), in the gills of juvenile *P. ornatus* after exposure to salinities: 25, 34, and 40 ppt between instar stages 7–9 (approx. 48 days, chronic exposure; *n* = 10, 12, 11). Mean (±SE). Boxplots show the median (horizontal line), interquartile range (box), and data spread (whiskers). Dots outside the whiskers represent outliers. Significant differences between treatments are indicated by different letters (*p* < 0.05).

Chronic salinity exposure significantly affected *nka* and *nkcc* gene expression (Figure 6A,B). Na^+^/K^+^-ATPase (*nka*) expression was higher at 25 ppt compared to 40 ppt but not elevated relative to the 34 ppt (Figure 6A, Table 3). Furthermore, salinity level had an effect on the expression of the *nkcc* gene (Figure 6C). Expression of *nkcc* was significantly higher at 25 ppt than at both 34 and 40 ppt. Relative expression of *nhe*, *nbc*, *ca* and *vhe* genes was unaffected by salinity level (Figure 6C–F and Table 3.

## 3. Discussion

Osmoregulation is important for the survival of marine crustaceans during periods of salinity change. *P. ornatus* is considered a weak hyper-osmoregulator, with a limited range of salinities below 34 ppt in which it can survive [4,15]. Understanding the physiological changes and adjustment to gene expression that occur during salinity response in *P. ornatus* juveniles can highlight environmental risks for the commercialisation of the species. The juveniles in this study weighed 6.23 ± 1.83 g and the selected instar stage matched the size of those first entering sea cage nursery culture in Vietnam [57].

### 3.1. Low- vs. Control Salinity Exposure

**Figure 7:**
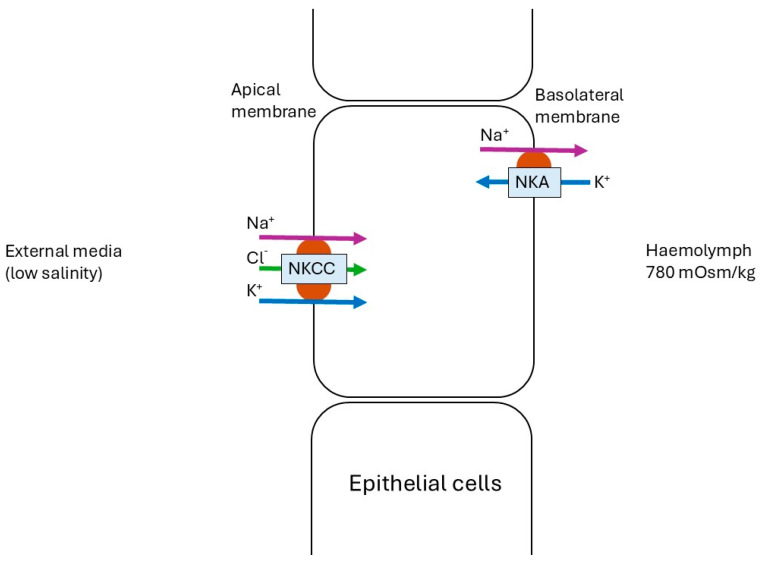
Coordinated function of Na^+^/K^+^-ATPase (NKA) = and Na^+^/K^+^/2Cl^−^ co-transporter (NKCC) in the epithelial cells of *P. ornatus* when exposed to low-salinity stress (25 ppt). A new theoretical framework was constructed for this study following the location of transporters reported in [17,29]. It should be noted that in a fish species, medaka *Oryzias dancena*, NKCC1a was positioned basolaterally in brackish water and seawater in medaka *Oryzias dancena*, and apically in freshwater-acclimated medaka [59]. Further studies are required to identify whether the cellular location of the NKCC in *P. ornatus* is apical, as shown here, or basolateral.

In this study, chronic exposure of juvenile *P. ornatus* to 25 ppt conditions resulted in increased expression of *nka* and *nkcc*. This pattern of expression following prolonged exposure indicates a transcriptional basis for maintaining high levels of NKA and NKCC activity during hyper-regulation, which has been seen in other crustacean species. For example, despite an initial decrease in NKA activity, there was a significant increase in mRNA expression after 1 h (6.5-fold) and at 5 and 24 h (3- to 4-fold) in the blue crab *Callinectes sapidus* cultured in 10 ppt salinity [58]. The increase in mRNA expression in *C. sapidus* appeared to support the subsequent rise in NKA activity observed after 24 h of exposure, suggesting the synthesis of new enzyme molecules [58]. Therefore, the elevation in expression of genes for NKA and NKCC in the chronically exposed gills in the present study suggests there was the continued and sustained need for these enzymes to be produced. At 25 ppt, juvenile *P. ornatus* haemolymph osmolarity was isosmotic with the external medium. Spencer, Fitzgibbon, Day, Trotter and Smith [15] reported that haemolymph osmolarity increased above ambient levels at salinities beyond the 25–34 ppt range, which is typically considered the osmoconforming range for most crustaceans. Therefore, homeostasis at 25 ppt is likely maintained by the coordinated activity of NKCC and NKA (Figure 7).

*Nkcc* was found to be significantly upregulated at 25 ppt, compared to both 34 and 40 ppt in chronic conditions, with a tendency towards upregulation following acute low-salinity exposure. Similarly to the present study, *nkcc* expression in mud crab, *Scylla paramamosain*, a euryhaline crustacean, was upregulated following exposure to low salinity (14 ppt) [60]. NKCC was also upregulated in the gills of another crab species, *Portunus trituberculatus*, following low-salinity (<34 ppt) exposure [61], although in that species, *nkcc* expression can increase during both high- and low-salinity acclimation at different times [37].The localisation of the NKCC in crustacean gills has not yet been identified and is a point for future research; however, models suggest a potential apical location in the crustacean gill [17,29,62], which is in opposition to the Na^+^ secretory role for the basolaterally located NKCC in the marine fish gill and salt-secretory glands from other animals [29]. Intriguingly, in the brackish medaka fish, *Oryzias dancena*, NKCC1a was positioned basolaterally in brackish water and seawater, and apically in freshwater-acclimated medaka [59]. Based on previous crustacean models [17,29], we are assuming that the upregulated *nkcc* of *P. ornatus* in low salinity was responsible for transporting Na^+^, K^+^, and Cl^−^ ions simultaneously from the external medium into the epithelial cell, playing a role in hyper-osmoregulation in low salinity.

Weak hyper-osmoregulators, like *P. ornatus*, have been previously suggested to lack mechanisms for active NaCl transport, allowing Na^+^ and Cl^−^ to diffuse passively and relatively freely across the cuticle and epithelium of the gill [17]. Therefore, for *P. ornatus* in low-salinity conditions, it is likely to be more reliant on passive rather than active ion transport mechanisms, most likely involving NKCC. Conversely, the expression of the four other genes identified to be involved in physiological responses to altered ambient salinity, *nbc*, *nhe vhe*, and *ca*, were found to be unaffected by chronic exposure to 25 ppt, which is likely due to their accessory enzyme roles and secondary functions. For instance, the lack of change in *vhe* expression following either acute or chronic exposure to low salinity is consistent with juvenile *P. ornatus* being considered stenohaline, with a limited range of salinity tolerance for survival [4,15]. These proteins of NBC, NHE, VHE and CA are more active in the gills of euryhaline than stenohaline marine species [63,64,65,66,67], and are salinity sensitive in euryhaline species [17]. Gill epithelia VHE secretes H^+^ out of the cell and into the external medium, creating a higher electrochemical gradient within the epithelial cells to assist with NaCl absorption across the gills [28,68]. Therefore, VHE is strongly associated with ionic regulation in strong osmoregulators, typically freshwater-adapted species [43,69,70]. The osmoregulation mechanism in freshwater species differs from that of marine species, with VHE being the driving force for ion flow during freshwater adaptation [43]. However, it is important to note that VHE may also contribute to cell volume regulation, and its functional role can vary depending on the external salinity. It could also be that the salinity level examined in this study was too high to see a change in *vhe* expression. Freshwater-adapted crustaceans, including the red crab *Dilocarcinus pagei* (hololimnetic) [71], Amazon river prawn, *Macrobrachium amazonicum* [72], and the aeglid crab *Aegla schmitti* (Anomura) [73], show increased enzyme activity following exposure to salinities below 20 ppt. Given that *P. ornatus* experiences high mortality below 25 ppt, the absence of additional osmoregulatory mechanisms prior to the salinity threshold at which VHE becomes active contributes to this vulnerability—particularly since the species is considered stenohaline.

Although NBC’s role in osmoregulation has been proposed [54], its specific function has not been previously described. Na^+^/HCO_3_^−^ exchanger (NBC) is an essential component of the acid/base homeostasis in crustaceans. The lack of effect on NBC during low-salinity challenges in this study may be linked to the channel’s heightened sensitivity to pH rather than salinity stress, with ambient pH remaining unaffected in the salinity challenges [55]. For instance, significant upregulation of NBC was detected following both low- and high-pH challenges in shrimp species [55]. Additionally, increased expression persisted longer following high-pH versus low-pH challenges, suggesting that NBC is more sensitive to high pH. Therefore, this study found no influence of salinity challenges on NBC, indicating that it likely plays a minimal role in the salinity adaptation of juvenile *P. ornatus*.

In crustaceans, the function of NHE is closely linked to that of NKA, with both transporters working together to regulate ion balance. During salinity adaptation in crustaceans, NKA drives Na^+^ transport into the haemolymph to maintain osmolarity, with apical Na^+^ uptake occurring via the NHE antiporter in freshwater-adapted species exposed to salinities below 25 ppt [16,23,27,73]. For example, during the larval stages (zoea V and decapodid) of the hyper-regulating palaemonid shrimp, giant river prawn *Macrobrachium pantanalense*, and Amazon river prawn *M. amazonicum*, expression of both NHE (isoform 3) and NKA was higher at 5 ppt than at 25 ppt [74]. Notably, *M. pantanalense*, a freshwater species, can hyper-regulate throughout its life cycle, whereas *M. amazonicum*, a brackish-water species, can only do so in first-stage larvae, late juveniles, and adults [74]. No difference in expression was observed between the two species during the juvenile stage at either salinity [74]. However, larval *M. pantanalense* exhibited significantly higher expression of both NHE and NKA than *M. amazonicum* at both salinities [74]. The lack of NHE expression change at 25 ppt in *P. ornatus* likely reflects its status as a weak hyper-osmoregulator, where NHE is not upregulated unless salinity drops below 25 ppt—the threshold at which the gene typically activates to support NKA-driven ion transport.

The lack of regulatory change in carbonic anhydrase (*ca*) expression in *P. ornatus* following either acute or chronic exposure to 25 ppt is unsurprising, given CA’s role as an accessory enzyme to NBC and NHE. Carbonic anhydrase (CA) plays a vital role in osmoregulation by catalysing the hydration of CO_2_, providing H^+^ and HCO_3_^−^ to power apical NHE, Cl^−^/HCO_3_^−^ exchangers, and VHE [28,75,76], and likely serves a similar function for NBC. In the ridge-tailed white prawn, *Exopalaemon carinicauda*, *ca* knockdown led to *nhe* downregulation and ultimately mortality under saline–alkaline stress [70]. Although *ca* expression is typically upregulated in euryhaline crustaceans with strong osmoregulatory capabilities, when exposed to low salinity [47,48,76,77,78,79], this response is absent in stenohaline species like *Cancer irroratus* [63]. As *P. ornatus* is considered a weak hyper-osmoregulator, it likely does not rely on CA for osmoregulation, which is consistent with the lack of responsiveness of its associated exchangers, NBC and NHE, to low-salinity (25 ppt) challenge.

Interestingly, in the preliminary investigation of the gill transcriptome of acutely exposed animals, one of the two DEGs highlighted from the comparison between 25 ppt and 34 ppt salinity was the putative fermi-chelate reductase 1. Its role is to convert insoluble ferric iron (Fe_3_^+^) into soluble ferrous iron (Fe_2_^+^) before it is transported from the endosome to the cytoplasm [80]. This gene was also found to be more highly expressed between low and high salinity. Iron is essential for the growth of aquatic animals, playing a crucial role in iron-sulphur proteins and enzymes, and participating in various metabolic processes such as deoxyribonucleic acid synthesis and repair, oxidative metabolism, and ecdysone synthesis [81,82,83,84]. Its potential role in the antioxidant capacity of juvenile *P. ornatus* is likely a cause of its increased expression following acute-salinity challenge. Considering the increased activity of NKCC and NKA, in juvenile *P. ornatus*, following low-salinity exposure, increased iron uptake could be a response to this heightened metabolic activity. Notably, this increased activity was not found to be harmful to juvenile *P. ornatus*, as no direct antioxidant enzyme response (superoxide dismutase) was observed in the haemolymph of individuals acutely [15] or chronically [4] exposed to either 25 or 40 ppt salinity in the previous literature. The role of iron in salinity adaptation has not yet been elucidated. However, it would be of interest for future research to investigate the potential role it plays.

### 3.2. High vs. Control Salinity Exposure

Expression of all investigated genes (*nka*, *nkcc*, *nhe*, *vhe*, *nbc*, and *ca*) remained unchanged following both acute and chronic exposure to 40 ppt salinity, relative to control conditions. This supports previous findings that active ion transport mechanisms are largely inactive above the critical salinity threshold (~26 ppt) in marine osmoregulators [17,25,60]. As salinity increases, gill NKA activity typically declines in some species, reflecting reduced hypo-regulatory capacity and limiting excess Na^+^ uptake under hyperosmotic conditions [25]. In the semi-terrestrial estuarine crab, *Chasmagnathus granulatus*, the *vhe*, *nka*, and *nkcc* expression remained unchanged during the first 96 h of exposure to 45 ppt, but increased exponentially thereafter, suggesting a delayed transcriptional response potentially linked to ion excretion [77]. Similarly, in the mud crab, *S. paramamosain*, *nka* mRNA expression was 3.5 times higher at 45 ppt than at 25 ppt, yet enzyme activity remained unchanged, indicating possible post-transcriptional regulation or enzyme degradation or loss of activity resulting from altered ambient ionic condition [34]. In contrast, *P. ornatus*, a weak hyper-osmoregulator, appears unable to activate these mechanisms under high salinity (>34 ppt), consistent with its limited capacity for active NaCl transport [17].

### 3.3. High- vs. Low-Salinity Exposure

NKA was significantly upregulated at 25 ppt compared with its expression at 40 ppt, with no difference compared to the control. Acting like a “gate,” NKA regulates ion flow across cell membranes, with a particularly vital role in gill epithelium [85]. Numerous studies have shown that *nka* gene expression increases during both short- and long-term low-salinity (<34 ppt) acclimation in marine crustacean gills [25,34,58,86,87], compensating for Na^+^ loss to the external medium via diffusion.

However, NKA is an active osmoregulatory mechanism that is metabolically demanding and plays a similar role in osmoregulation to NKCC in osmoconforming crustaceans such as *P. ornatus*. This study found that the specific growth rate (SGR) and moult increment length of juvenile *P. ornatus* at 40 ppt were significantly lower than at 25 and 34 ppt. Similarly, Spencer, Fitzgibbon, Day, Trotter and Smith [4] reported that juvenile *P. ornatus* exposed to 40 ppt for 75 days had a significantly lower SGR compared to those maintained at 25, 30, and 34 ppt. Interestingly, the scalloped spiny lobster, *Panulirus homarus*, showed no adverse effects after six weeks at 38 ppt, likely due to increased expression of NADH dehydrogenase in the gills [88,89]. Most of the DEGs at 38 ppt in this species were related to energy metabolism [88,89]. As a coastal species not typically found in brackish environments, it is speculated that 38 ppt falls within the tolerable salinity range for *P. homarus*, and that gene expression is modulated to mitigate salinity stress [88].

Following the initial examination of the transcriptome, it is important to highlight that in both high- and low-salinity-exposed *P. ornatus gill samples*, the genes for antioxidant enzymes were upregulated. Glutathione S-transferase (GST) was significantly higher in the RNA-Seq samples of 25 ppt-exposed juveniles than in 40 ppt-exposed ones, while glutathione peroxidase (GPx) was upregulated in the high-salinity-exposed juveniles. However, neither were significantly different compared to the control exposure of 34 ppt. Both GST and GPx are involved in the antioxidant system, utilising glutathione to protect the cells from free radical damage. The antioxidant superoxide dismutase, which precedes GPx in the reaction chain, was unaffected by exposure to 40 ppt in juvenile *P. ornatus* following either acute or chronic exposure [4,15]. Metabolic pathways involved in energy metabolism, including GST, were upregulated in the transcriptome of the *S. paramamosain* at 3‰ salinity compared to 23‰ in 24 h [90]. Therefore, GST likely responds to the increased metabolic demands of NKA, indicating higher metabolic rates during lower-salinity conditions compared to high salinity, but not exceeding control levels.

Interestingly, an ammonia transporter was also upregulated in the 40 ppt salinity transcriptomes compared to the 25 ppt salinity transcriptomes. Crustaceans have been known to increase ammonia excretion during 25 ppt salinity (hyperregulation) but not 40 ppt salinity (hypo-regulation) exposure [91,92,93,94]. For instance, following acclimation to five salinities between 18 ‰ and 42 ‰, kuruma prawn and *Marsupenaeus japonicus* showed that ammonia-N excretion and nitrite-N excretion decreased with increasing salinity [95]. Spencer, Fitzgibbon, Day, Trotter and Smith [4] theorised that reduced growth at 40 ppt was likely due to the species’ limited osmoregulatory capacity, leading to cell dehydration and death under prolonged high-salinity exposure. The unexpected upregulation of ammonia transporters may reflect a breakdown in cellular function, where increased ammonia excretion demands exacerbate water loss and elevate haemolymph osmolarity. This could be further intensified by water loss at osmoregulatory sites such as the antennal gland or gut, which warrants future investigation. It should be reiterated, although the findings suggest potential trends, the limited replication under high-salinity conditions limits the strength of the conclusions and warrants cautious interpretation.

In general, the key osmoregulatory genes examined in this study were not significantly different between 34 and 40 ppt. This supports the hypothesis proposed in Spencer, Fitzgibbon, Day, Trotter and Smith [4], indicating that *P. ornatus* lacks salinity-sensitive ionic transport mechanisms, either passive or active, during periods of hyper-osmotic (>34 ppt) stress.

### 3.4. Acute vs. Chronic Exposure Times

NKA and NKCC were found to be significantly upregulated only during chronic exposure to 25 ppt, while all other key osmoregulatory genes were unaffected by any salinity treatment. This suggests NKA and NKCC are the only ion-transporting mechanisms in juvenile *P. ornatus* under hypo-saline conditions, but only during prolonged exposure. However, the timing of exposure needs to be considered as an influencing factor. For *S. paramamosain* [60], *nkcc* mRNA was downregulated during the first 24 h of exposure to 40 ppt, then returned to pre-exposure levels around 48 to 72 h later. Similarly, for the burrowing crab, *C. granulatus*, it was not until 96 h of exposure to 45 ppt that mRNA expression of *nkcc* increased and remained at this level for the remaining 8 d of the experiment [77]. Considering the acute response time interval in the present study was no less than 48 h, it is likely that the downregulation of *nkcc* could have been missed during acute examination, particularly any downregulation of the genes of interest following high-salinity stress. For instance, Cl/HCO_3_^−^ expression (though not directly examined in this study) has a fast turnover rate, with significant decreases in Lv-AE3 transcripts observed at 3 h under high- and low-salinity challenges [96]. Future research should consider shorter time intervals, with 48 h being the minimum time checked for salinity influence on gene expression.

### 3.5. Implication on Aquaculture

This research highlights the importance of understanding species-specific osmoregulatory capacities prior to initiating aquaculture production. Euryhaline species, such as the pacific white shrimp, *Litopenaeus vannamei* species, are well-established in open pond culture [97]. In contrast, this study finds that the stenohaline species *Panulirus ornatus* is physiologically incapable of tolerating salinity fluctuations beyond the range of 25 to 34 ppt, supported by molecular data. This suggests that culturing *P. ornatus* in outdoor pond systems in tropical regions would be challenging. High mortality rates are likely throughout the year due to seasonal monsoons and heatwaves, which can cause prolonged and repeated fluctuations in pond salinity [98]. To mitigate these risks, measures such as pond covers to reduce the impact of heavy rainfall and access to freshwater during heatwaves (which may be limited during drought conditions) would be essential.

### 3.6. Study Limitation and Future Prospects

One of the primary limitations of this study was the limited number of transcriptome biological replicates, particularly under 40 ppt salinity conditions. As a result, the transcriptomic analysis should be considered a preliminary investigation into gene expression responses to acute salinity stress. While the data provide valuable initial insights, the limited replication restricts the statistical power and generalise ability of the findings.

Despite this, the transcriptomic data were critical in identifying and confirming the sequences of six key genes of interest in this species, laying the groundwork for future functional and expression studies. To strengthen the conclusions drawn and validate the observed expression patterns, future research should incorporate a greater number of biological replicates across all treatment conditions. This will allow for more robust statistical analyses and a clearer understanding of the molecular mechanisms underlying salinity adaptation.

The acute 40 ppt salinity-exposed sample, which was removed from statistical analysis due to variation, was likely from an individual in the pre-moult stage. Spencer, Fitzgibbon, Day, Trotter and Smith [15] demonstrated differences in salinity tolerance across moult-cycle stages, with the pre-moult stage being the most sensitive to low-salinity exposure. For future investigations, the effects of moult stage and life stage on gene expression should be considered.

Due to the potential for time-dependent changes in gene expression, future studies should consider a broader range of time intervals to assess the expression dynamics of the six genes examined in this study.

Due to budgetary constraints, the study was limited in its ability to explore additional time frames for acute salinity exposure and to include a larger number of individuals per salinity treatment. Expanding these parameters would allow for a more comprehensive examination of gene expression patterns related to osmoregulation in *P. ornatus* following salinity adaptation.

Future research into the salinity adaptation of spiny rock lobsters should also investigate other tissues, such as the antennal glands. These glands are considered to play an important role in osmoregulation yet remain largely unexplored. Since the acute samples were obtained from a prior experiment, antennal glands were not extracted, and therefore, tissues other than the gills could not be examined.

Finally, future research should investigate potential changes in animal physiology and behaviour associated with the differential expression of these genes in response to varying salinity levels. This would contribute to a clearer understanding of the relationship between genotypic responses and observable phenotypic traits.

## 4. Methods

*P. ornatus* juveniles were reared from eggs and cultured to juveniles at the University of Tasmania’s Institute for Marine and Antarctic Studies (IMAS), Hobart Aquaculture Facility following previously established protocols [99,100]. Prior to experimental treatments, juvenile lobsters were communally reared in 18 L polyethylene vessels at densities of 200 individuals per m^2^ at a water temperature of 28 ± 0.1 °C and a 12:12 h (light:dark) photoperiod. Lobsters were fed 3% of their body weight twice daily with an IMAS Commercial in Confidence formulated lobster feed, once in the morning and in the evening. Vessels were syphon-cleaned and checked for moulting events prior to the morning feed. All *P. ornatus* used for this study were between instar 7 and 9 (between 7th and 9th moult post-puerulus) and in the intermoult period as a representative size of lobsters that are normally caught for seeding lobster aquaculture operations in Vietnam for onshore grow-out systems [57]. The acutely exposed samples were collected between 5 and 10 days after moulting, during instar stages 7 to 9. They were subsequently exposed for 48 h. The chronic exposed samples were all collected 5 days post-moult to instar stage 9. A five-day period post-moult was allowed to ensure that the lobsters’ exoskeletons had fully hardened and that each individual was firmly within the inter-moult stage prior to the commencement of the experiment, thereby reducing variability associated with moulting physiology. Due to individual variation in moult timing, this corresponded to an exposure duration of approximately 38 to 52 days.

### 4.1. Acute Exposure

Lobster were sampled following methods of Spencer, Fitzgibbon, Day, Trotter and Smith [15], whereby the left and right gills were collected separately from 12 individual juvenile *P. ornatus* between instar stages of 7–9 (5.13 ± 1.85 g) exposed for 48 h to one of three salinity treatments 25, 34, and 40 ppt (*n* = 4 per treatment). The samples were flash-frozen in liquid nitrogen and stored at −80 °C prior to RNA extraction. The samples from acutely exposed animals were used to generate transcriptomes and were analysed for the purpose of determining the presence of osmoregulatory genes of interest in *P. ornatus* juveniles, prior to commencing with qPCR work on chronic cultured gill samples.

### 4.2. Chronic Exposure

#### 4.2.1. Experimental System and Protocol

Following moulting to instar phase J7 + 3 days (2.8 ± 0.8 g), *P. ornatus* juveniles (*n* = 36) were exposed to one of three salinity treatments: 25, 34 and 40 ppt, with each salinity treatment consisting of 12 replicate lobsters (*n* = 12). The lobsters were weighted (WW) and had their carapace length (CL) measured prior to being stocked at a density of three per tank, across sixteen 18 L rectangular polyethene vessels (0.390 m length × 0.245 m width × 0.255 m height). Vessels received water from one of three salinity-independent RAS systems (as described below) at a water flow rate of six exchanges per h^−1^ and contained an air stone to maintain dissolved oxygen (DO). Since lobster moulting is not synchronised, replicate stocking was randomly staggered, with treatment commencement once each replicate was fully stocked with three lobsters. To prevent cannibalisation, lobsters were separated by two plastic partitions positioned diagonally across the corners of the tank, dividing the tank into four equal sections. The partitions had 1.5 mm wide vertical slots (125 mm high, starting 40 mm off the tank floor) to permit water flow, but restricted cannibalistic behaviour. The one section of each tank not holding an animal, contained an air stone and the outflow screen. Water inflow was provided to each section housing a lobster to ensure adequate flow throughout the experimental vessel. Each lobster was provided a hide (40 mm and 555 mm diameter by 50 mm length polyvinyl chloride pipes) lined with 5 mm oyster mesh. Following each moulting event, lobsters’ WW and CL were measured in the morning after a moulting event. Vessel maintenance was as previously described, and lobsters were fed daily with 3% of their body weight with 1.2 mm IMAS Commercial in Confidence formulated lobster feed pellets once every 3 h overnight (2100 to 0600 h).

#### 4.2.2. Acclimation Period

Prior to stocking into experimental vessels, lobsters were acclimated in 1.5 L cylindrical vessels. Each acclimation vessel contained 1.5 L of saline water adjusted to 5 ppt below or above the control salinity of 34 ppt, except for control lobsters, which were maintained at 34 ppt throughout the acclimation period. Animals were held in these conditions for 24 h, with a complete (100%) water exchange performed at the 12 h mark. Water quality parameters during acclimation were maintained at the conditions as described: temperature 27.0 ± 0.3 °C, pH 8.12 ± 0.03, dissolved oxygen 102 ± 2% saturation, and ammonia < 0.25 ppm NH_4_^+^. The photoperiod was set to a 12:12 h light:dark cycle. Following acclimation, lobsters were manually transferred to experimental vessels for chronic exposure to their designated salinity treatments.

#### 4.2.3. RAS System

To maintain water quality, each RAS was equipped with a 70 L trickle biofilter sump, a 9 W UV lamp (Eheim, Clear UVC7) and 10 μm mesh filter bag. Every seven days, each RAS system received a 50% (250 L) water exchange with treatment water. When salinity exceeded the target level by more than 1 ppt, systems were diluted using 1 μm filtered freshwater (Siemens Memcor membrane filtration, Windsor, Australia). Salinity treatment water was prepared in 350 L batches following the methods of Spencer, Fitzgibbon, Day, Trotter and Smith [15]. For the 25 ppt treatment, 1 μm filtered seawater was diluted with filtered freshwater. For the 40 ppt treatment, marine salt (Red Sea Salt, R11065) was added to filtered seawater. Salinity was verified using a WTW Multi 3430 (Weilheim, Germany) probe. Control systems, maintained at 34 ppt, received a 50% water exchange with filtered seawater every seven days. Each systems temperature was maintained at 27.5 ± 0.3 °C by a heating unit (TECOPONIC tk-3000^®^, https://www.tecoponics.com/tk-3000 (accessed on 10 February 2023). Culture vessel pH and DO were measured daily using a water quality probe (WTW Multi 3430) and maintained at DO = 101% ± 2 and pH = 8.15 ± 0.03. Ammonia and nitrite concentrations were measured before each 50% water exchange using a Hach DR1900 spectrophotometer (Hach Pacific, Melbourne, Australia) with nitrogen–ammonia (HACH 2604545) and nitrogen–nitrite (HACH 2605345) reagent sets. Average concentrations across the study were 0.02 ± 0.04 mg/L NH_3_-N and 0.02 ± 0.005 mg/L NO_2_^−^-N. Lighting conditions during the experiment matched those used during prior culture (12:12 h light:dark cycle).

#### 4.2.4. Sampling

Juveniles were terminally sampled once they were at J9 + five days. This ensured that the lobsters were no longer considered post-moult and were in the most stable phase of the crustaceans’ inter-moult cycle [101,102]. Animals were euthanised by being put in 0 °C treatment saline water for 60 s. Each lobster was individually placed in a fresh 2 L bath of treatment salinity water to prevent cross-contamination between animals. The WW and CL were recorded before dissection.

A total of 30 µL of haemolymph was collected via cardiac puncture using a pre-chilled, sterile 0.3 mL 31G insulin needle inserted beneath the carapace. The haemolymph was centrifuged at 800× *g* for 3 min in a pre-chilled 1.5 mL Eppendorf tube. The resulting plasma was pipetted into pre-chilled cryotubes, flash-frozen in liquid nitrogen, and stored at −80 °C for subsequent osmolarity analysis.

Following haemolymph being drawn, both the left and right gill-set were extracted separately and snap-frozen in liquid nitrogen. Samples were preserved at −80 °C until taken on dry ice from IMAS to the University of the Sunshine Coast (UniSC), for RNA extraction. Samples were stored at −80 °C at UniSC until RNA extraction.

During the experiment, three mortalities occurred: two in the 25 ppt treatment, both related to moulting, and one in the 40 ppt treatment, unrelated to moulting.

### 4.3. RNA Extraction

Total RNA was extracted separately from both the right and left gill-sets of 12 acutely exposed lobsters and 33 chronically exposed lobsters using RNAzolRT (Sigma, Melbourne, Australia) with the addition of 1% *v*/*v* β-mercaptoethanol, following methods previously described by Lewis et al. [103], with the exception being that mechanical homogenisation was carried out using an Omni tissue rotor–stator homogeniser (TH) (Omni International, Melbourne, Australia). RNA extracted was quantified using a NanoDrop 2000 instrument (ThermoFisher, Melbourne, Australia) and kept at −80 °C.

### 4.4. Gill Transcriptome Sequencing, Assembly and Analysis: Acute Salinity Exposure

To assess differences in gene expression in the gills following acute (48 h) salinity exposure, nine gill samples (three individuals per salinity treatment, *n* = 3), were selected for RNA sequencing. Due to cost constraints, the three highest-quality RNA samples from each treatment group were used for transcriptome analysis, based on RNA integrity and concentration values. A minimum of 3 µg RNA for each of the nine gill-sets were desiccated in Ambient RNA tubes (Novogene) and sent to Novogene (Singapore) for quality control, followed by library preparation (TrueSeq) and RNA Sequencing using Illumina Novaseq PE150 (paired end 150 bp). FastQ files of between 4 and 5.8 Gb for each sample were generated (at least 60 million reads per sample). CLC Genomics Workbench 8.0.3 (Qiagen, Australia) was used for trimming, assembly and RNAseq analysis. Reads were trimmed (five nucleotides from the 5’ end) with reads less than 80 bp discarded. Reads from one animal per treatment were de novo assembled with minimum contig lengths of 500 nucleotides, producing 45,818 contigs. Trimmed reads from each sample were mapped to the gill assembly using a similarity fraction of 0.8 which gave approximately 70% mapping rate per sample. Reads per kilobase per million reads (RPKM) values were calculated for each lobster sample. Principal component analysis (PCA) was carried out using CLC to examine similarity in all gene expression between samples.

Differential expression of transcripts between each salinity pair was filtered using the parameters: fold change (absolute value) > 2, FDR *p* value correction < 0.05 and differentially expressed genes identified for each comparison were characterised. The Empirical analysis of DGE algorithm was used (CLC Genomics Workbench). The Expasy translate tool (https://web.expasy.org/translate/ (accessed on 11 November 2023)) was used to translate the contig nucleotide sequences and to identify the likely coding sequence of each gene. Translated sequences were subjected to BLAST searches using the NCBI nr database (https://www.ncbi.nlm.nih.gov/ (accessed on 11 November 2023)) to characterise the differentially expressed genes. Since some genes coded for uncharacterised proteins, the translated sequences were put into the SMART http://smart.embl-heidelberg.de/ (accessed on 12 December 2023) website to determine protein domains and to provide information on potential functions for these proteins in the gill.

The sequences of six genes of interest (*nka*, *nkcc*, *ca*, *vhe*, *nhe*, and *nbc*) were identified through a BLAST search (http://www.ncbi.nlm.nih.gov/ (accessed on 11 November 2023)) of known protein sequences of osmoregulatory genes in other decapod crustaceans. The functional roles of several gene isoforms were evaluated by examining the previous literature on crustacean osmoregulation under salinity stress [25,32,34,50,72,86,104,105,106,107,108,109,110,111,112,113,114] of crustacean osmoregulation under salinity stress. The *P. ornatus* sequences were identified by conducting a BLAST search of known sequences against the gill transcriptomes from all salinity treatments in the present study, as well as a *P. ornatus* transcriptome database comprising adult tissues and developmental life stages in CrustyBase, https://crustybase.org/ [115,116]. Open reading frames for the transcripts of interest were determined using expasy (https://www.expasy.org/), followed by domain annotation via SMART [117] to further validate transcript identity. To confirm the identity of our genes of interest, a phylogenetic approach was employed with known sequences from other model decapods retrieved from NCBI (See Table S1 for accession numbers). Amino acid sequences were aligned with MAFFT (https://doi.org/10.1093/nar/gkf436) using the FFT-NS-2 setting [118] in using the Galaxy Australia [119] webserver. The alignment was then trimmed with ClipKIT (https://doi.org/10.1371/journal.pbio.3001007) using the smart-gap trimming mode, and a maximum likelihood tree was constructed using IQ tree (https://doi.org/10.1093/molbev/msu300). Model finder was used to predict the best fitting model (JTTDCMut+F+R4) and 10,000 bootstrap replications were performed. The final tree was exported in Newick format to ITOL (https://itol.embl.de/ (accessed on 6 February 2024)) for annotation. (Figures S2–S7).

The RPKM values for each transcript determined the expression of the six genes of interest in the gills and allowed a comparison to be made between the lobsters acutely exposed to the three different salinities.

### 4.5. Quantitative PCR (qPCR) Analysis of Osmoregulatory Gene Expression: Chronic Salinity Exposure

Following transcriptome analysis of gene expression after acute exposure to different salinities, the expression of the genes of interest was quantified in the gills of animals chronically exposed to the different salinities using qPCR.

The RNA extracted from the gills of the juvenile lobsters (*n* = 12) exposed to one of three salinities for a chronic period (38–52 days) was prepared for qPCR analysis. Synthesised cDNA was produced from 1 µg of each RNA sample using a Tetro cDNA kit (Meridian Bioscience, Melbourne, Australia). qPCR was performed to detect the expression levels of genes coding for VHE, CA, NKA, NHE, NKCC, and NBC in the gills of chronically exposed animals. The alpha-subunit was targeted in the primers of both NKA and the beta-subunit in VHE. The sequence for 18S rRNA was used as an internal reference gene. Polymerase chain reaction amplification was performed using MyTaq polymerase HS mix (Bioline, Meridian Bioscience) mixed with SYBR, and the 10 µL reaction mix contained, 5 µL of MyTaq, 0.5 µL of both the left and right primer, 2 µL of molecular water and 2 µL of DNA template. The qPCR was run on a Bio Rad CFX connect Real-time system. The following programme was used for PCR amplification: initial denaturation at 95 °C for 3 min, followed by 40 cycles at 95 °C for 10 s, 55 °C for 25 s, 72 °C for 25 s, with a final elongation at 95 °C for 5 min. A melting-curve analysis was added to the end of each PCR reaction to verify the specificity of the product. Primers were designed using Primer 3, and domain locations were the primary target of their design (Table 4).

All the samples for qRT-PCR analysis were prepared in triplicates. Following qPCR analysis, the delta Ct (ddCt) values for each sample was then calculated using 18S results as a reference gene. Relative expression was calculated using the 2^(ddCt)^ method, as previously described [120]. The mean relative expression for each treatment was subsequently calculated for following statistical analysis.

### 4.6. Osmolarity

The osmolarity was recorded in triplicates of 10 µL of heamolymph plasma; osmolarity was measured using a vapour osmometer (VAPRO pressure osmometer, model 5520).

### 4.7. Statistical Analysis

Statistical analyses were conducted with RStudio version 2023.06.02+561. Shapiro–Wilk’s and Barlett’s tests were used to test for normality and homogeneity of variance in the data, respectively. One-way ANOVA with a Tukey HSD post hoc analysis was used to compare data, including salinity impact on Initial WW, Final WW, Final CL, WW gain, CL gain, moult growth increment (J8–J9) and moult increment (J8–J9). Values from multiple one-way ANOVAs were adjusted using the Benjamini–Hochberg procedure to account for multiple comparisons across the six genes. For data found to be not normally distributed and without equal variance, which included all genes of interest for both acute and chronic salinity-exposed gills, a Kruskal–Wallis one-way analysis of variance was used. Post hoc pairwise comparisons following a significant Kruskal–Wallis test were conducted using Dunn’s test, with *p*-values adjusted for multiple comparisons across the six genes using the Benjamini–Hochberg procedure. Osmolarity was examined through a Gaussian family generalised linear model (GLM). A post hoc ‘emmeans’ function with a Tukey adjustment was used to further investigate significant associations between treatments. Survival was converted into a percentage of the total number of survivors for each salinity treatment. The J7 stage was excluded from the moult growth and moulting increment analyses due to stress-induced and asynchronous moulting. Stress-induced moulting refers to a moulting event which occurs due to a stressor such as handling. Moulting increment is the time length in d between J8–J9. Moult growth increment is the measured specific growth rate between J8 and J9 stages. Moult growth increment was calculated by

$$Moult\;growth\;increment\;\left(\%BW\;d^{-1}\right)=\frac{\left(In\;final\;J9\;weight\;\left[g\;WW\right]-In\;J8\;weight\;\left[g\;WW\right]\right)*100}{number\;of\;d\;between\;moulting\;events\;}$$

## 5. Conclusions

In line with previous physiological studies, this study confirms *P. ornatus* as a stenohaline weak hyper-osmoregulator. Its weak osmoregulatory capacity stems from limited expression of metabolically active transporter enzymes and key ion channels required to generate favourable NaCl gradients. For the first time, the importance of the Na^+^/K^+^/2Cl^−^ cotransporter (NKCC) has been identified in the gills of *P. ornatus* exposed to low salinity (25 ppt), across both chronic and acute time frames. Although NKCC is not an ATPase, its function is energetically dependent on gradients established by Na^+^/K^+^-ATPase, which was found to be upregulated at low salinity to help maintain haemolymph osmolarity. *P. ornatus* lacks mechanisms to manage salinities above its ambient range, and exposure to elevated salinities leads to reduced growth between moulting events and high mortality rates if prolonged. Determining the molecular mechanisms behind salinity adaptation in *P. ornatus* provides crucial insight into its potential for onshore aquaculture development. This knowledge is essential for optimising aquaculture practices and improving culture efficiency.

## Figures and Tables

**Table 1:** Survival (%), initial and final wet weight (WW, g) and carapace length (CL, mm), moult growth increment (J8–J9, %BWd^−1^) and moult increment (days) of *P. ornatus* juveniles reared at different salinities (ppt) for two-moult increments. Mean (±SE), 25 ppt: *n* = 10, 34 ppt: *n* = 12 and 40 ppt: *n* = 11. **df** = degrees of freedom; **F/χ^2^** = test statistic (F-value for ANOVA or χ^2^ for Kruskal–Wallis test); **P** = *p*-value indicating statistical significance (typically, *p* < 0.05 is considered significant). * indicates significant differences present and significant differences between means within the same row are distinguished by different letters in superscript.

Salinity (ppt)	25	34	40	df	F or x^2^	P	Test
Survival (%)	83	100	91				
Initial WW (g)	2.88 ± 0.89	3.20 ± 0.91	2.84 ± 0.45	1, 40	0.015	0.91	ANOVA
Initial CL (mm)	11.5 ± 1.98	11.3 ± 1.88	12.0 ± 2.47	3	1.14	0.77	Kruskal–Wallis
Final WW (g)	5.90 ± 1.028	7.02 ± 1.83	5.79 ± 1.34	1, 33	0.012	0.91	ANOVA
Final CL (mm)	21.58 ± 1.93	22.22 ± 2.11	20.22 ± 2.73	1, 33	1.331	0.257	ANOVA
WW gain (g)	3.57 ± 1.06	3.82 ± 0.99	2.95 ± 1.38	1, 33	1.16	0.29	ANOVA
CL gain (mm)	18.1 ± 5.71	14.0 ± 1.42	8.98 ± 8.03	3, 14	2.19	0.15	ANOVA
Moult growth increment (%BWd^−1^) (J8–J9)	7.65 ^ab^ ± 2.43	8.89 ^b^ ± 2.67	5.19 ^a^ ± 2.16	2, 33	7.267	0.01 *	ANOVA
Moult increment (days) (J8–J9)	18.7 ^a^ ± 3.62	19.93 ^a^ ± 3.75	26.0 ^b^ ± 7.09	2, 33	6.823	0.01 *	ANOVA

**Table 2:** Preliminary analysis of branchial Na^+^/K^+^-ATPase (*nka*), V-type H^+^-ATPase (*vhe*), Na^+^/HCO_3_^−^ exchanger (*nbc*), Na^+^/K^+^/2Cl^−^ co-transporter (*nkcc*), Na^+^/H^+^ exchange protein (*nhe*), and carbonic anhydrase (*ca*) gene expression in *P. ornatus* juveniles following an acute (48 h) salinity exposure (25, 34 and 40 ppt). Mean (±SE), 25 ppt: *n* = 3, 34 ppt: *n* =3 and 40 ppt: *n* = 2. Total expression (RPKM). **df** = degrees of freedom; **χ^2^** = test statistic (χ^2^ for Kruskal–Wallis test); **P** = *p*-value indicating statistical significance (typically, *p* < 0.05 is considered significant).

Salinity (ppt)	25	34	40	df	x^2^	P	Test
NKA (RPKM)	481.9 ± 144.35	248.8 ± 129.30	233.65 ± 46.46	2	4.03	0.39	Kruskal–Wallis
CA (RPKM)	803.37 ± 223.67	811.30 ± 294.08	608.55 ± 185.47	2	0.47	0.88	Kruskal–Wallis
VHE (RPKM)	31.13 ± 5.85	33.66 ± 6.21	33.45 ± 12.94	2	0.25	0.88	Kruskal–Wallis
NKCC (RPKM)	235.43 ± 183.12	7.03 ± 5.51	0.60 ± 0.45	2	6.33	0.15	Kruskal–Wallis
NBC (RPKM)	107.03 ± 28.02	70.90 ± 26.76	48.40± 10.04	2	3.14	0.42	Kruskal–Wallis
NHE (RPKM)	11.90 ± 1.32	10.76 ± 4.83	8.60 ± 0.28	2	2.47	0.43	Kruskal–Wallis

**Table 3:** Relative gene expression (ddCt) of branchial Na^+^/K^+^-ATPase (*nka*), V-type H^+^-ATPase (*vhe*), Na^+^/HCO_3_^−^ exchanger (*nbc*), Na^+^/K^+^/2Cl^−^ co-transporter (*nkcc*), Na^+^/H^+^ exchange protein (*nhe*), and carbonic anhydrase (*ca*) from *P. ornatus* juveniles communally reared at different salinities (ppt) for two-moult stages (J7–J9). Mean (± SE), 25 ppt: *n* = 10, 34 ppt: *n* = 12 and 40 ppt: *n* = 11. **df** = degrees of freedom; **χ^2^** = test statistic (χ^2^ for Kruskal–Wallis test); **P** = *p*-value indicating statistical significance (typically, *p* < 0.05 is considered significant). * indicates significant differences present and significant differences between means within the same row are distinguished by different letters in superscript.

Salinity (ppt)	25	34	40	df	x^2^	P	Test
NBC (ddCt)	3.56 ± 3.22	3.12 ± 2.35	1.95 ± 1.31	2	2.61	0.32	Kruskal–Wallis
CA (ddCt)	11.83 ± 16.40	7.96 ± 8.71	3.29 ± 3.34	2	3.40	0.27	Kruskal–Wallis
NKA (ddCt)	9.03 ^a^ ± 7.30	3.98 ^ab^ ± 3.37	2.27 ^b^ ± 1.55	2	10.35	>0.01 *	Kruskal–Wallis
VHE (ddCt)	5.27 ± 4.06	17.63 ± 40.93	3.28 ± 1.97	2	0.88	0.65	Kruskal–Wallis
NHE (ddCt)	3.93 ± 2.62	2.73 ± 1.52	1.62 ± 0.99	2	6.64	0.07	Kruskal–Wallis
NKCC (ddCt)	283.65 ^a^ ± 224.43	6.34 ^b^ ± 4.71	5.94 ^b^ ± 14.48	2	21.50	>0.01 *	Kruskal–Wallis

**Table 4:** Primer sequences.

Gene Name	Primer Sequence	Sequence	Amplicon Size
V-type H^+^-ATPase	Forward	TGTTTGCGGTCATGTTTGGT	241
	Reverse	CGTATGTGCCAAGATGAGCC	
carbonic anhydrase	Forward	TGGCACTCCTCGTTCAAGAT	208
	Reverse	CGCATGATAGAGGGAGGTGT	
Na^+^/K^+^-ATPase	Forward	TAACTCTCACAGCCAAGCGA	235
	Reverse	GAAAGTGCCTTCCAGCCTTC	
Na^+^/H^+^ antiporter	Forward	TGCCAACAACAACCACAACT	242
	Reverse	TGGCAGGTCAACACTCGTAT	
Na^+^/K^+^/Cl^−^ cotransporter	Forward	GGAGGGTATGCTGATCGTGA	210
	Reverse	ACAGAAGCCCACGATGTACA	
18s	Forward	AACGGACTTGACGGTTGGTT	21
	Reverse	CTGTTCGGAGCCTGACAGAA	
Na^+^/HCO_3_^−^ co-transporter	Forward	ACTCAGGGTCAGGCTTCTTC	116
	Reverse	GCGTGTAATGGAGCCAGATG	

## Data Availability

The transcriptomic data presented in this study can be found on CrustyBase.org, accessed on 17 December 2023. The data has also been uploaded to the NCBI sequence read archive under the following BioProject number: PRJNA1356957. Transcripts DEG annotations and phylogenetic tree accession numbers have been made available in the Supplementary Material (Tables S1 and S2). Physiological and qPCR data will be made available on request.

## Supplementary Materials

The following supporting information can be downloaded at https://www.mdpi.com/article/10.3390/ijms262211150/s1.
