# Supplementary material for: Assessing Molecular Mechanisms of Stress Induced Salinity Adaptation in the Juvenile Ornate Spiny Lobster, Panulirus ornatus
Source: Int J Mol Sci. 2025 Nov 18;26(22):11150. doi: 10.3390/ijms262211150 (PMC12652530; doi:10.3390/ijms262211150)
Supplement: Supplementary file 1 [file ijms-26-11150-s001.zip › ijms-3962364-supplementary/Supplementary Figure S1.pdf]

A

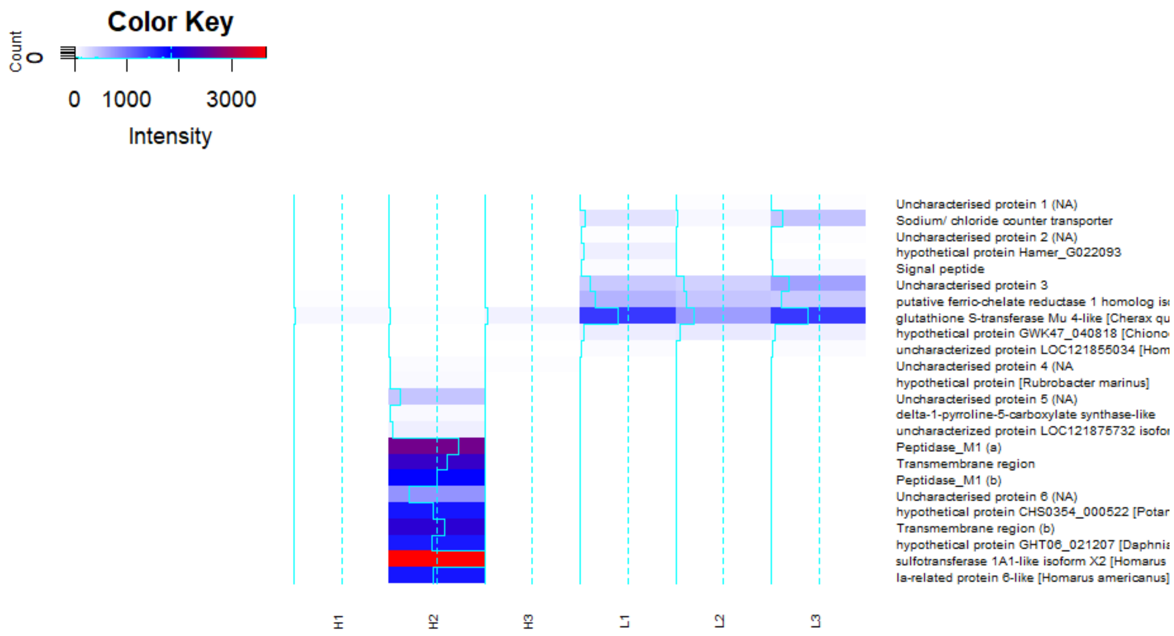

B

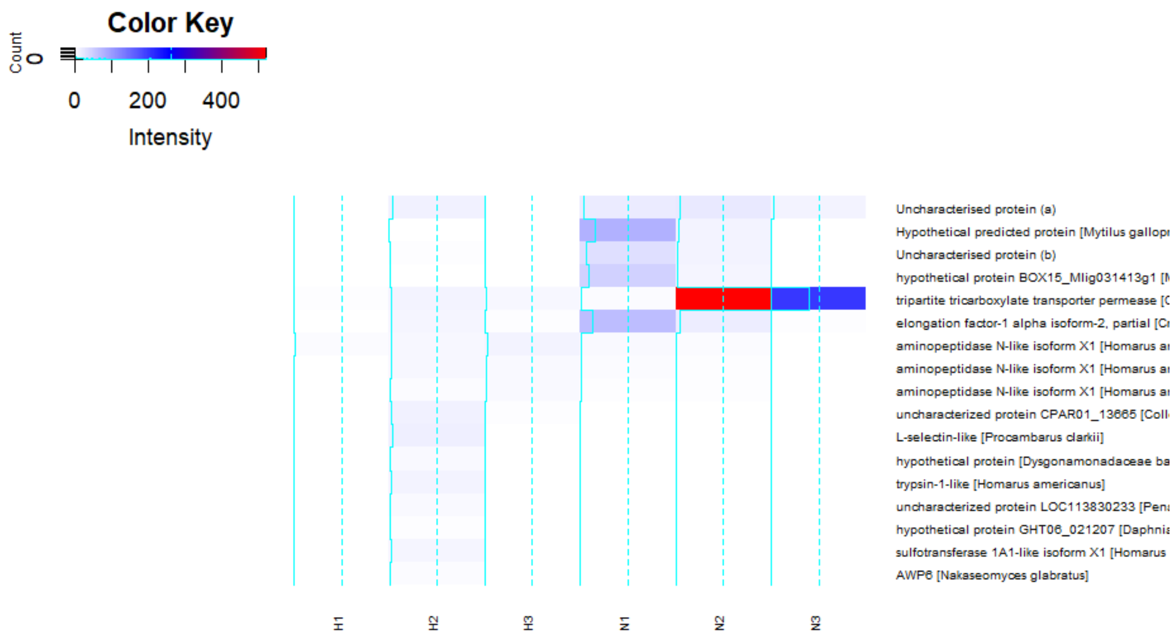

Figure S1: Initial transcriptome analysis heatmap of gills exposed to three salinity treatments (25, 34 and 40 ppt) in juvenile (instar 7- 9) *Panulirus ornatus* for 48 h (acute exposure).

Differentially expressed genes only are shown (FDR < 0.05, fold-change > 2). A: Low (25 ppt, L) vs high (40 ppt, H) salinity, B: Control (34 ppt, N) vs high (40 ppt, N) salinity.. 25 ppt: N = 3, 34 ppt: N=3 and 40 ppt: N = 2., B). The intensity of the transcript expression, represented as RPKM, are displayed as colours ranging from white (lowest expression) to dark red (highest expression). Notably, sample H2 displayed markedly higher RPKM values compared to the other high salinity (40 ppt) samples, which disproportionately influenced the initial differential expression analysis. This anomaly raised concerns prior to PCA testing of samples and was a key factor in the decision to exclude H2 from subsequent analyses.
