# Supplementary material for: Assessing Molecular Mechanisms of Stress Induced Salinity Adaptation in the Juvenile Ornate Spiny Lobster, Panulirus ornatus
Source: Int J Mol Sci. 2025 Nov 18;26(22):11150. doi: 10.3390/ijms262211150 (PMC12652530; doi:10.3390/ijms262211150)
Supplement: Supplementary file 1 [file ijms-26-11150-s001.zip › ijms-3962364-supplementary/Supplemtary Figures. S1-S7 .pdf]

Supplementary figures.

A

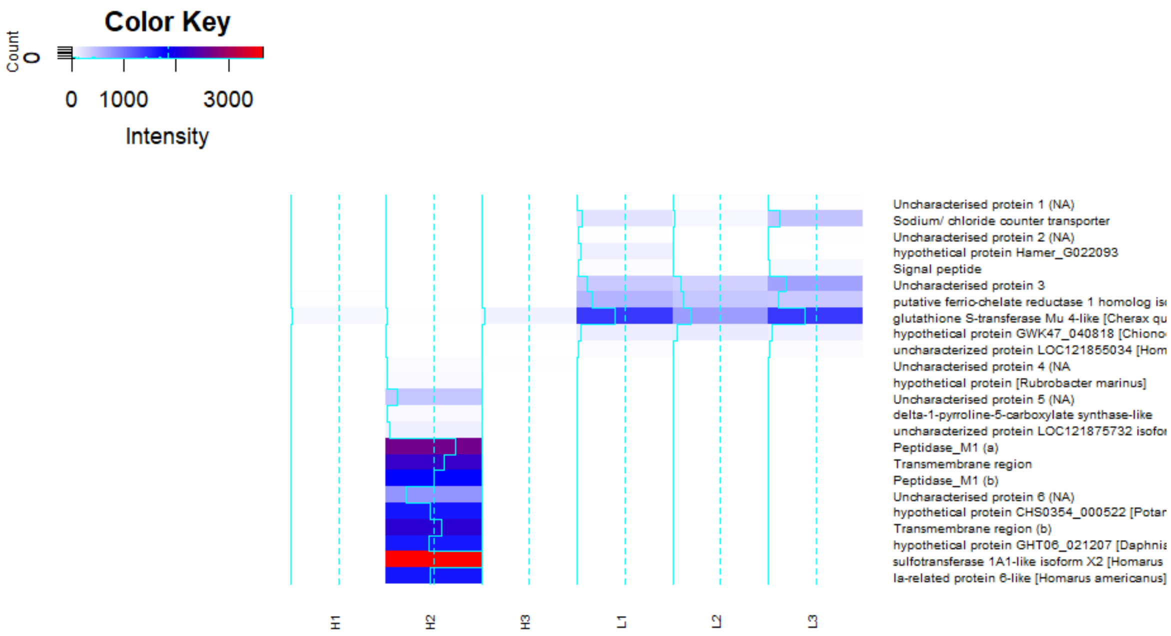

B

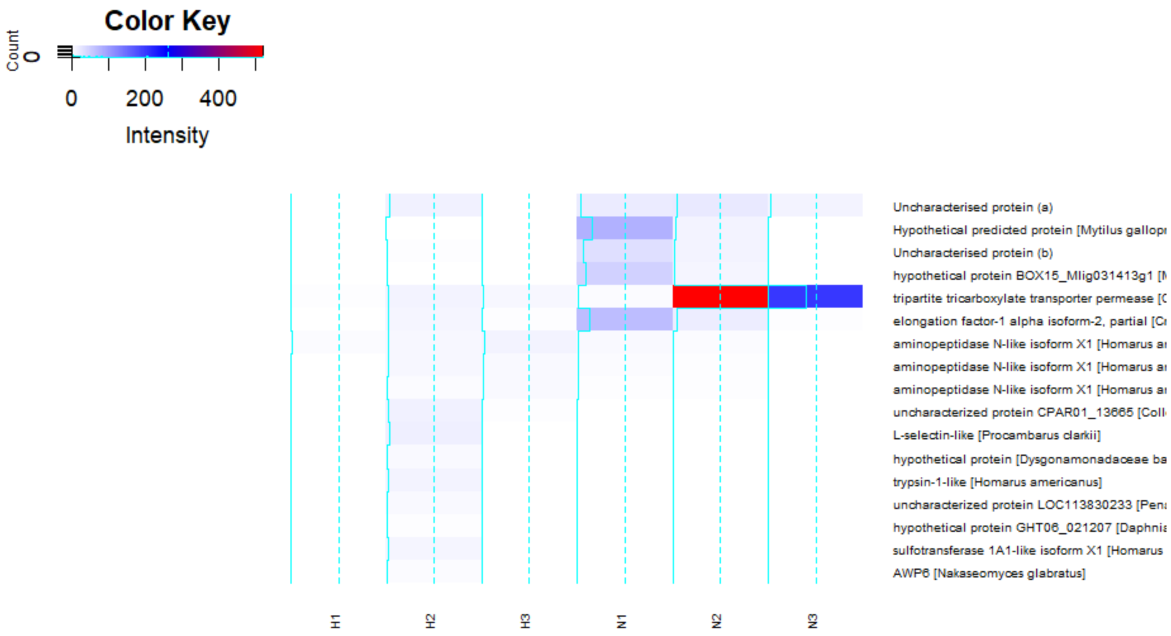

Figure S1: Initial transcriptome analysis heatmap of gills exposed to three salinity treatments (25, 34 and 40 ppt) in juvenile (instar 7- 9) *Panulirus ornatus* for 48 h (acute exposure). Differentially expressed genes only are shown (FDR < 0.05, fold-change > 2). A: Low (25 ppt, L) vs high (40 ppt, H) salinity, B: Control (34 ppt, N) vs high (40 ppt, N) salinity.. 25 ppt: N = 3, 34 ppt: N=3 and 40 ppt: N = 2., B). The intensity of the transcript expression, represented as RPKM, are displayed as colours ranging from white (lowest expression) to dark red (highest expression). Notably, sample H2 displayed markedly higher RPKM values compared to the other high salinity (40 ppt) samples, which disproportionately influenced the initial differential expression analysis. This anomaly raised concerns prior to PCA testing of samples and was a key factor in the decision to exclude H2 from subsequent analyses.

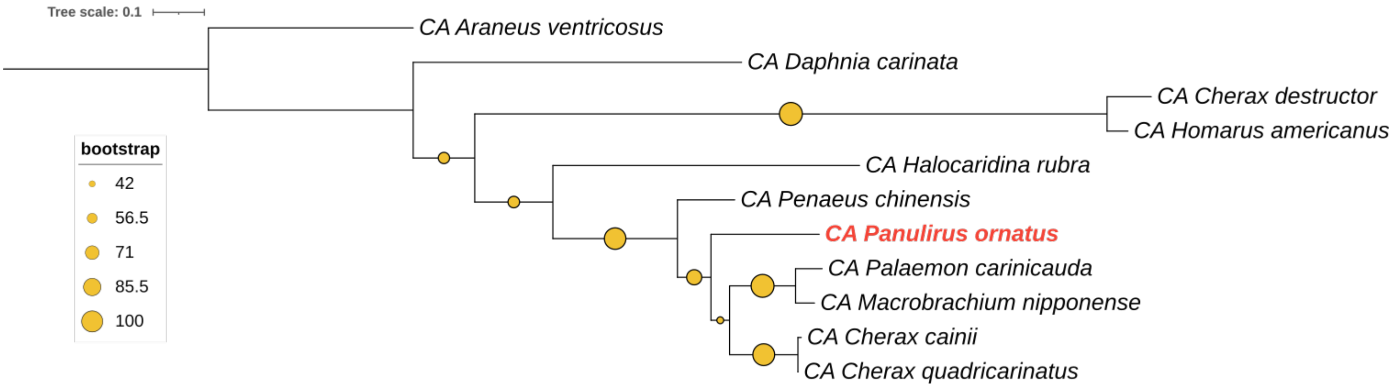

Figure S2. Maximum likelihood tree of identified Carbonic anyhydrase (CA), *transcript from Panulirus ornatus alongside other model decapod sequences retrieved from NCBI. Amino acid sequences were aligned with MAFFT, trimmed with ClipKIT, and a maximum likelihood tree was constructed with IQ-tree predicting the best fit substitution model* WAG+I+G4*) with ModelFinder. Bootstrap values (10000 replicates) are indicated by yellow circles, where size is scaled to bootstrap percentages. Putative transcripts identified in P. ornatus are highlighted red and bolded, and other decapod species include Cherax destructor, Homarus americanus, Halcoaridina rubra, Penaeus chinensis, Cherax cainii, Cherax quadricarinatus, Palaemon carinicauda, Macrobrachium nipponense, Daphnia carinata, with Areneus centricosus (an insect) CA as roots.*

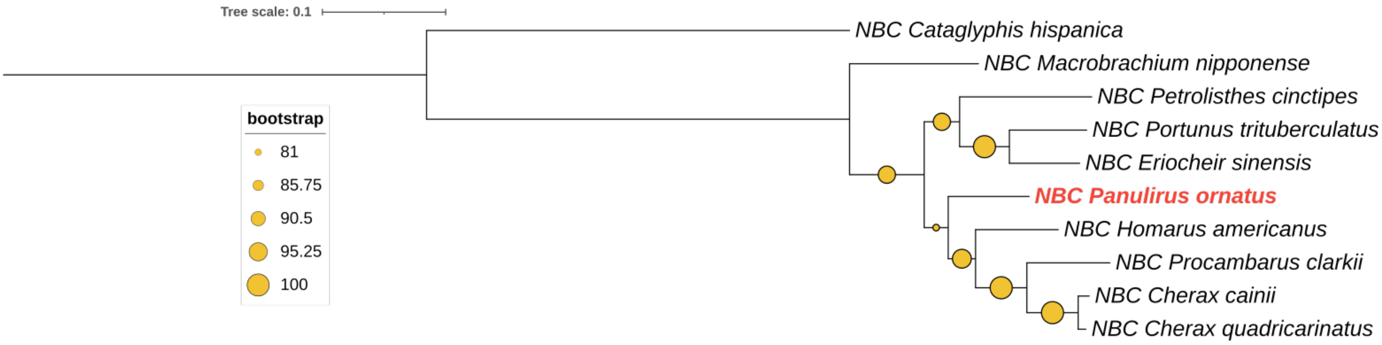

Figure S3. Maximum likelihood tree of identified Na+/HCO3- exchanger (NBC)*, transcript from Panulirus ornatus alongside other model decapod sequences retrieved from NCBI. Amino acid sequences were aligned with MAFFT, trimmed with ClipKIT, and a maximum likelihood tree was constructed with IQ-tree predicting the best fit substitution model* JTTDCMut+F+R3*) with ModelFinder. Bootstrap values (10000 replicates) are indicated by yellow circles, where size is scaled to bootstrap percentages. Putative transcripts identified in P. ornatus are highlighted red and bolded, and other decapod species include Portunus trituberculatus, Erocheir sinesis, Macrobrachium nipponense, Homarus americanus, Procambarus clarkii, Cherax cainii, Charax quadricarinatus, Petrolisthes cinctipes, with Cataglyphis hispanica (an insect) NBC as a root*

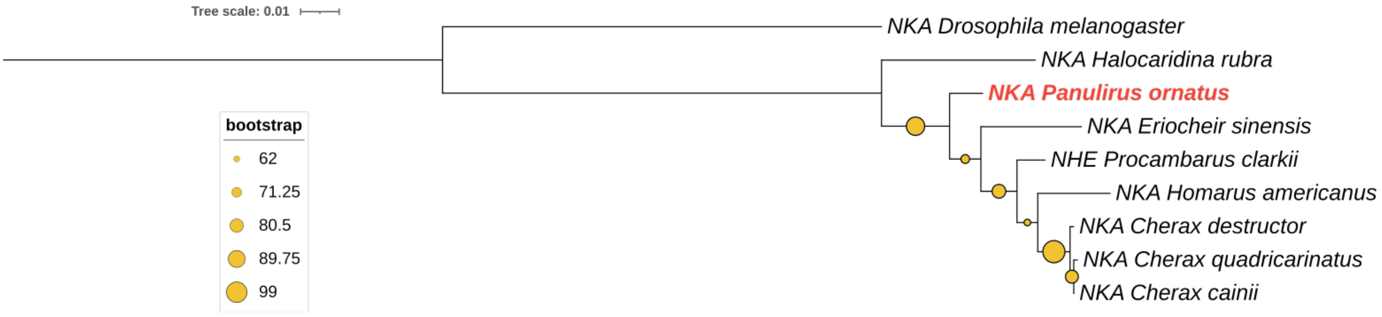

Figure S4. Maximum likelihood tree of identified Na+/K+-ATPase (NKA), transcript from Panulirus ornatus alongside other model decapod sequences retrieved from NCBI. Amino acid sequences were aligned with MAFFT, trimmed with ClipKIT, and a maximum likelihood tree was constructed with IQ-tree predicting the best fit substitution model LG+I+G4*) with ModelFinder. Bootstrap values (10000 replicates) are indicated by yellow circles, where size is scaled to bootstrap percentages. Putative transcripts identified in P. ornatus are highlighted red and bolded, and other decapod species include Halocaridina rubra, Eriocheir sinesis, Procambarus clarkii, Homarus americanus, Cherax destructor, Cherax quadricarinatus, Cherax cainii, Amadillidium nasatum with Drosophila melanogaster (an insect) NKA as a root.*

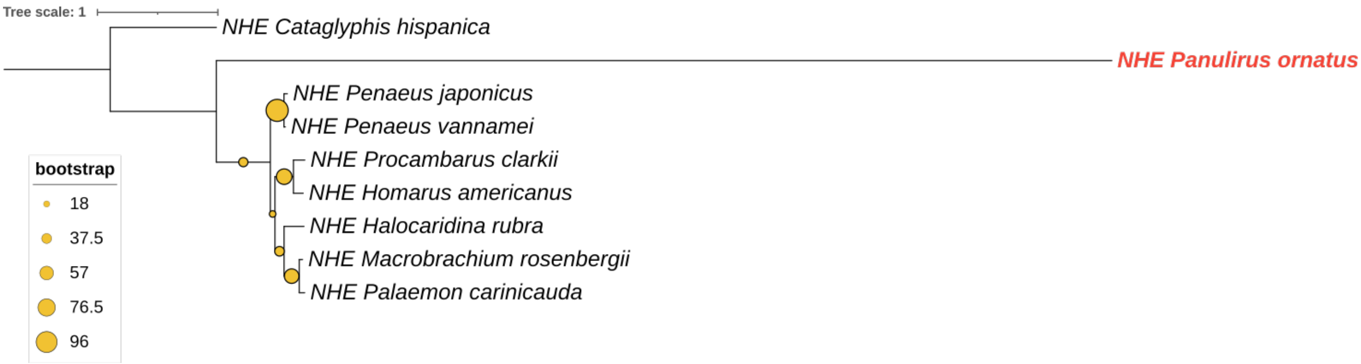

Figure S5. Maximum likelihood tree of identified Na+/H+ exchange protein (NHE), *transcript from Panulirus ornatus alongside other model decapod sequences retrieved from NCBI. Amino acid sequences were aligned with MAFFT, trimmed with ClipKIT, and a maximum likelihood tree was constructed with IQ-tree predicting the best fit substitution model* JTT+F+I+R3*) with ModelFinder. Bootstrap values (10000 replicates) are indicated by yellow circles, where size is scaled to bootstrap percentages. Putative transcripts identified in P. ornatus are highlighted red and bolded, and other decapod species include Procambarus clarkii, Homarus americanus, Penaeus japonicus, Penaeus vannamei, Halocardiuna rubra, Palaemon carinicauda, Macrobrachium rosenbergii , with Cataglyphis hispanica (an insect) NHE as a root*

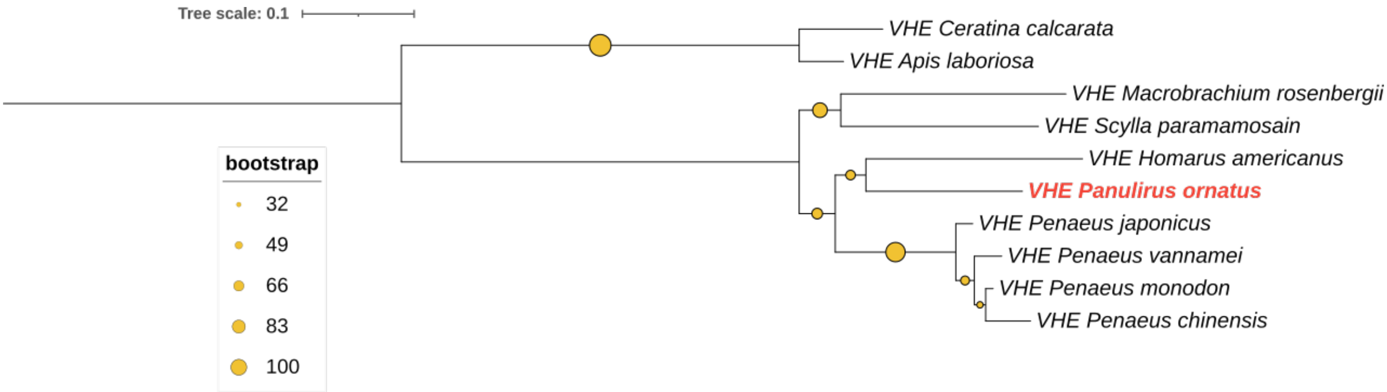

Figure S6. Maximum likelihood tree of identified V-type H+-ATPase (VHE), *transcript from Panulirus ornatus alongside other model decapod sequences retrieved from NCBI. Amino acid sequences were aligned with MAFFT, trimmed with ClipKIT, and a maximum likelihood tree was constructed with IQ-tree predicting the best fit substitution model* LG+I+R2*) with ModelFinder. Bootstrap values (10000 replicates) are indicated by yellow circles, where size is scaled to bootstrap percentages. Putative transcripts identified in P. ornatus are highlighted red and bolded, and other decapod species include Homarus americanus, Macrobrachium rosenbergii, Scylla paramamosain, Penaeus chinesis, Penaeus monodon, Penaeus japonicus, Penaeus vannamei, Ceratina calcarata and Apis laboriosa (an insect) VHE as root.*

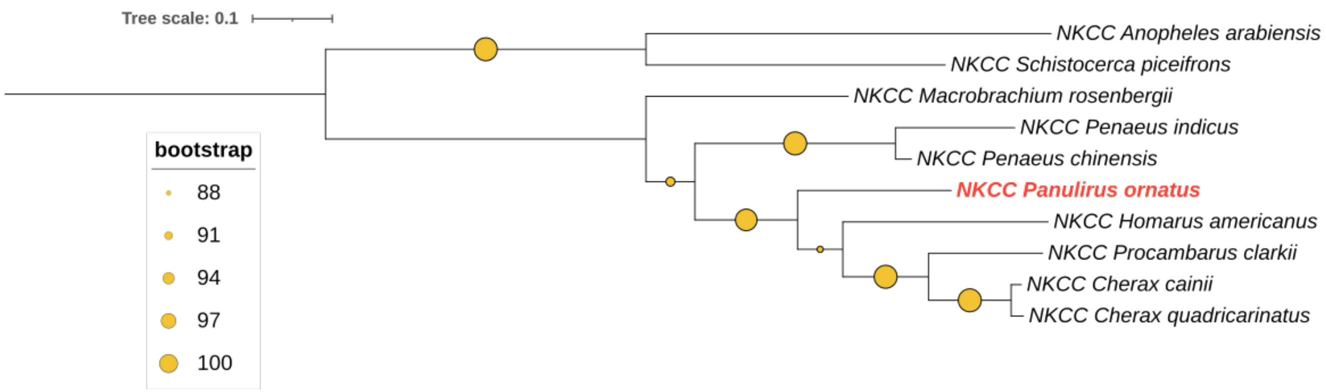

Figure S7: Maximum likelihood tree of identified Na+/K+/2Cl- co-transporter (NKCC), transcript from Panulirus ornatus alongside other model decapod sequences retrieved from NCBI. Amino acid sequences were aligned with MAFFT, trimmed with ClipKIT, and a maximum likelihood tree was constructed with IQ-tree predicting the best fit substitution model LG+F+I+R3*) with ModelFinder. Bootstrap values (10000 replicates) are indicated by yellow circles, where size is scaled to bootstrap percentages. Putative transcripts identified in P. ornatus are highlighted red and bolded, and other decapod species include Macrobrachium rosenbergii, Penaeus indicus, Penaeus chinesis, Homarus americanus, Procambarus clarkii, Cherax cainii, Cherax quadricarinatus, Schistocerca piceifrons with Anopheles arabiensis (an insect) Schistocerca piceifrons (an insect) NKCC as roots.*
